# Supplementary material for: Measurement characteristics and correlates of HIV-related stigma among adults living with HIV: a cross-sectional study from coastal Kenya
Source: BMJ Open. 2022 Feb 22;12(2):e050709. doi: 10.1136/bmjopen-2021-050709 (PMC8867337; doi:10.1136/bmjopen-2021-050709)
Supplement: Supplementary data [file bmjopen-2021-050709supp001.pdf]

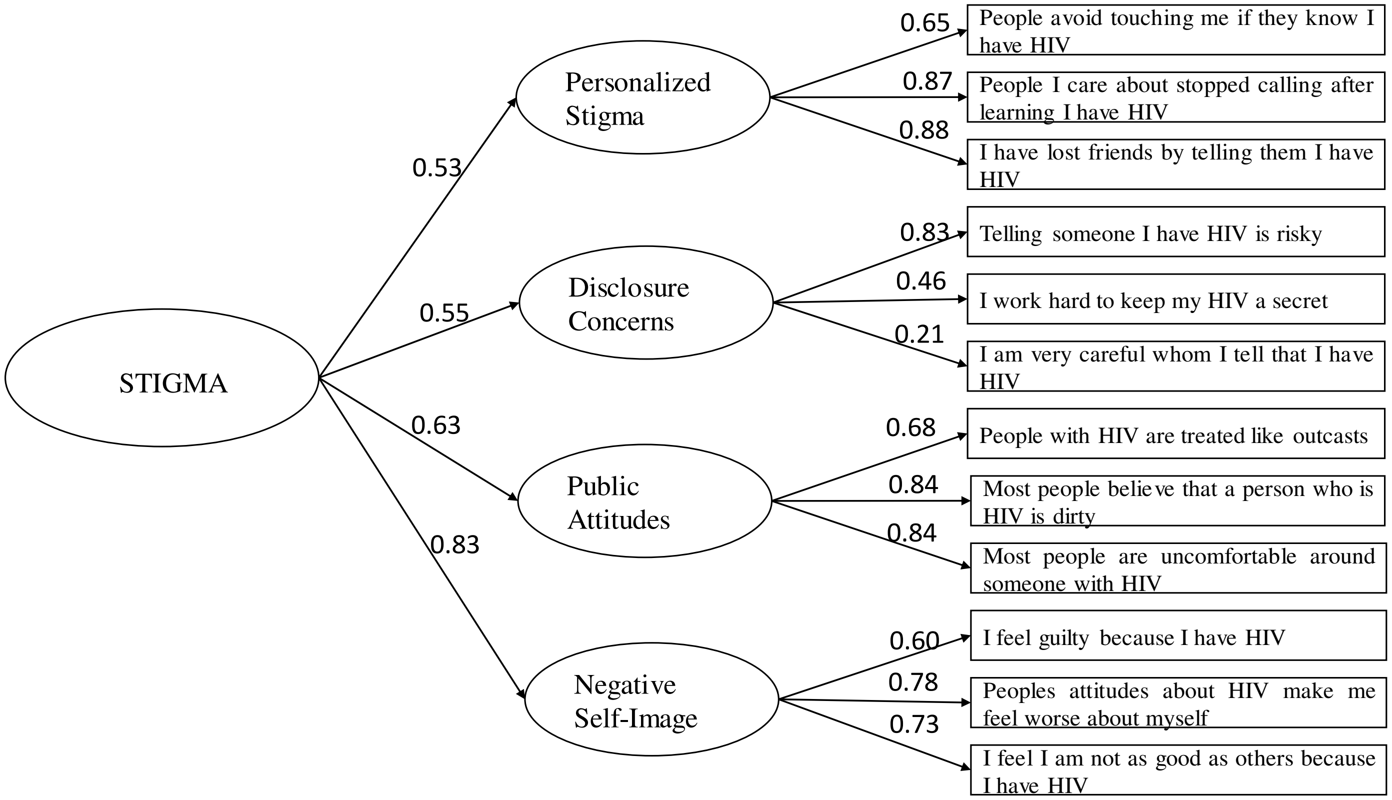

Supplementary Figure 1: Confirmatory factor analysis of the short version of the HIV Stigma Scale. Results show correlations between subscales (circles) and maximum likelihood estimates for the relation between subscales and items (rectangles). Sample (n = 435). Maximum likelihood estimates are standardised
