# Supplementary material for: Measurement characteristics and correlates of HIV-related stigma among adults living with HIV: a cross-sectional study from coastal Kenya
Source: BMJ Open. 2022 Feb 22;12(2):e050709. doi: 10.1136/bmjopen-2021-050709 (PMC8867337; doi:10.1136/bmjopen-2021-050709)
Supplement: Author's manuscript [file bmjopen-2021-050709.draft_revisions.pdf]

BMJ Open

BMJ Open is committed to open peer review. As part of this commitment we make the peer review history of every article we publish publicly available.

When an article is published we post the peer reviewers' comments and the authors' responses online. We also post the versions of the paper that were used during peer review. These are the versions that the peer review comments apply to.

The versions of the paper that follow are the versions that were submitted during the peer review process. They are not the versions of record or the final published versions. They should not be cited or distributed as the published version of this manuscript.

BMJ Open is an open access journal and the full, final, typeset and author-corrected version of record of the manuscript is available on our site with no access controls, subscription charges or pay-per-view fees (http://bmjopen.bmj.com).

If you have any questions on BMJ Open's open peer review process please email info.bmjopen@bmj.com

BMJ Open

HIV-Related Stigma: Measurement Characteristics and Correlates among Adults Living with HIV at the Kenyan Coast

Journal:	*BMJ Open*
Manuscript ID	bmjopen-2021-050709
Article Type:	Original research
Date Submitted by the Author:	02-Mar-2021

Complete List of Authors:	Wanjala, Stanley; Pwani University School of Humanities and Social Sciences, Social Sciences; Ghent University Faculty of Medicine and Health Sciences, Public Health and Primary Care<br>Nyongesa, Moses; Centre for Geographic Medicine Research Coast, Clinical Neurosciences; Vrije Universiteit Amsterdam, Clinical, Neuro- and Developmental Psychology, Amsterdam Public Health Research Institute<br>Mwangi, Paul; Centre for Geographic Medicine Research Coast, Clinical Neurosciences<br>Mutua, Agnes M.; Centre for Geographic Medicine Research Coast, Clinical Neurosciences<br>Luchters, Stanley; Ghent University Faculty of Medicine and Health Sciences, Public Health and Primary Care; Aga Khan University - Kenya, Population Health<br>Newton, Charles; Centre for Geographic Medicine Research Coast, Clinical Neurosciences; Oxford University, Psychiatry<br>Abubakar, Amina A.; Centre for Geographic Medicine Research Coast, Clinical Neurosciences; The Aga Khan University - Kenya, Institute for Human Development

Keywords:	HIV & AIDS < INFECTIOUS DISEASES, Epidemiology < INFECTIOUS DISEASES, PUBLIC HEALTH

SCHOLARONE™ Manuscripts

I, the Submitting Author has the right to grant and does grant on behalf of all authors of the Work (as defined in the below author licence), an exclusive licence and/or a non-exclusive licence for contributions from authors who are: i) UK Crown employees; ii) where BMJ has agreed a CC-BY licence shall apply, and/or iii) in accordance with the terms applicable for US Federal Government officers or employees acting as part of their official duties; on a worldwide, perpetual, irrevocable, royalty-free basis to BMJ Publishing Group Ltd ("BMJ") its licensees and where the relevant Journal is co-owned by BMJ to the co-owners of the Journal, to publish the Work in this journal and any other BMJ products and to exploit all rights, as set out in our licence.

The Submitting Author accepts and understands that any supply made under these terms is made by BMJ to the Submitting Author unless you are acting as an employee on behalf of your employer or a postgraduate student of an affiliated institution which is paying any applicable article publishing charge ("APC") for Open Access articles. Where the Submitting Author wishes to make the Work available on an Open Access basis (and intends to pay the relevant APC), the terms of reuse of such Open Access shall be governed by a Creative Commons licence – details of these licences and which Creative Commons licence will apply to this Work are set out in our licence referred to above.

Other than as permitted in any relevant BMJ Author's Self Archiving Policies, I confirm this Work has not been accepted for publication elsewhere, is not being considered for publication elsewhere and does not duplicate material already published. I confirm all authors consent to publication of this Work and authorise the granting of this licence.

For peer review only

HIV-Related Stigma: Measurement Characteristics and Correlates among Adults Living with HIV at the Kenyan Coast

Stanley W. Wanjala[1,2*], Moses K. Nyongesa[3,4], Paul Mwangi[3], Agnes M. Mutua[3], Stanley Luchters[2,5,6,7], Charles R.J.C. Newton[3,8,9], Amina A Abubakar[3,8,9,10]

[1]Department of Social Sciences, Pwani University, Kilifi, Kenya

[2]Department of Public Health and Primary Care, Ghent University, Ghent, Belgium

[3]Neuroassessment Group, KEMRI/Wellcome Trust Research Programme, Centre for Geographic Medicine Research (Coast), Kilifi, Kenya.

[4]Department of Clinical, Neuro- & Developmental Psychology, Vrije Universiteit Amsterdam, Amsterdam, Netherlands

[5]Burnet Institute, Melbourne, Australia

[6]Department of Epidemiology and Preventive Medicine, Monash University, Melbourne, Australia

[7]Department of Population Health, Aga Khan University, Nairobi, Kenya

[8]Department of Public Health, Pwani University, Kilifi, Kenya

[9]Department of Psychiatry, University of Oxford, Oxford, UK

[10]Institute for Human Development, Aga Khan University, Nairobi, Kenya

Address of the corresponding author: Stanley W. Wanjala, Department of Social Sciences Pwani University, Box 195-80108, Kilifi, Kenya. Email: s.wanjala@pu.ac.ke

Target journal: BMJ Open

Word count: 4695

For peer review only

For peer review only - http://bmjopen.bmj.com/site/about/guidelines.xhtml

Abstract (*300 words*)

Objective We studied the psychometric properties of the 12-item short version of the Berger HIV stigma scale and assessed the correlates of HIV-related stigma among adults living with HIV on the Kenyan coast.

Design Cross-sectional study.

Setting Comprehensive Care and Research Centre in the Kilifi County Hospital.

Participants Adults living with HIV and on combination antiretroviral therapy were recruited and interviewed between February and April 2018 (n=450).

Main outcome measures HIV related stigma

Results 450 participants with a median age of 43 years (IQR = 36-50) took part in the study. Of these, 356 (79.1%) were female. Scale reliability and validity were high (alpha=0.80, test-retest reliability intraclass correlation coefficient =0.92). Using confirmatory factor analysis, we observed that the 12-item short version of the HIV stigma scale had a good fit for its hypothesised model (Comparative Fit Index =0.966, Tucker Lewis Index = 0.955, Root Mean Square Error of Approximation = 0.044). Multi-group confirmatory factor analysis indicated measurement invariance across gender and age groups as ΔCFI was ≤0.01. Multivariate linear regression established that being female *(β=2.001, 95%CI: 0.21, 3.80, p= 0.029),* HIV status non-disclosure *(β=4.237, 95%CI: 1.27, 7.20, p= 0.005)* and co-occurrence of depressive and anxiety symptoms *(β=6.670, 95%CI: 3.40, 9.94, p<0.001)* were significant predictors of perceived HIV- related stigma and that these variables accounted for 10.2% of the explained variability in HIV-related stigma among adults living with HIV from Kilifi.

Conclusions Our results indicate that the 12-item short version of the HIV stigma scale is a valid and reliable measure of HIV stigma in Kenya. Furthermore, our study indicates that interventions aimed at reducing stigma need to take into account gender to address the specific needs of women, people who have not disclosed their HIV status, and those exhibiting symptoms of depression and anxiety, thereby improving their quality of life.

Keywords: Adults**,** Stigma, Predictors, HIV/AIDS, antiretroviral therapy, Psychometrics, Kenya

For peer review only - http://bmjopen.bmj.com/site/about/guidelines.xhtml

Article Summary

Strengths and limitations of this study

- This is the first study to report the 12-item HIV stigma scale's measurement characteristics in the sub-Saharan African context.
- We report on the correlates of HIV stigma based on a culturally adapted measurement tool with good psychometric properties.
- We cannot generalise our findings to all adults living with HIV in Kenya as data were collected from one geographical setting and excluded adults older than 60 years.
- We cannot conclude how individuals experience stigma over time because of the study design limitation.

For peer review only

For peer review only - http://bmjopen.bmj.com/site/about/guidelines.xhtml

Introduction

HIV/AIDS remains a considerable public health concern globally, with sub-Saharan Africa (SSA) bearing the most HIV-related disease burden.[1] Despite SSA making up about 11% of the earth's population, it is the world's epicentre of HIV/AIDS. By the close of 2019, an estimated 38 million people were living with HIV globally, with an estimated 68% living in SSA, accounting for two-thirds of all HIV infected individuals.[1] Estimates show that between 80% to 90% of the people living with HIV/AIDS (PLWHA) in Kenya are adults.[2] Between 2010 and mid-2020, there has been an upsurge in the number of people accessing antiretroviral therapy (7.8- 26 million). Further, between 2010 and 2019, new HIV infections declined by an estimated 16% from 2.1 Million/year to 1.7 million/year, and AIDS-related deaths dropped from 1.1 million to around 690,000 per year.[1]

Erving Goffman[3] defined stigma as a process through which individuals are 'disqualified from full social acceptance' due to an undesirable 'mark' or 'label.' This label can either be a physical, health, or behavioural attribute that is regarded as 'deeply discrediting.'[3] In this study, the label is HIV seropositive status. Additionally, stigma, defined as a 'mark,' sets a person apart from others and links the person to undesirable characteristics such as stereotypes.[4] HIV-related stigma among PLWHA is prevalent throughout SSA.[5] HIV-related stigma has been identified as a severe obstacle in the way of effective responses to HIV.[6]

Although efforts have been scaled up to raise awareness and increase public knowledge about HIV since the epidemic started decades ago, social stigma is still associated with the disease.[7] Research has demonstrated that stigma keeps people from adopting HIV preventive behaviours and accessing needed care and treatment[8], negatively impacting their health and well-being. Among HIV-infected women, the decision to disclose their HIV seropositive status is likely affected by perceived stigma.[9]

From previous research, HIV stigma experienced by PLWHA can either be enacted, anticipated, or internalised.[10] Enacted stigma includes an individual's experiences, prejudice, and/or discrimination from others because of one's HIV status. Anticipated stigma includes an individual's expectation of experiencing enacted stigma, while internalised stigma refers to the extent to which PLWHA have adopted negative feelings and beliefs about PLWHA.[11]

A variety of instruments designed to measure HIV-related stigma have been published.[12-20] Berger's 40-item HIV stigma scale (HSS-40) is the most commonly used instrument and one of the few instruments covering all stigma mechanisms affecting PLWHA.[11] It takes up to 25

For peer review only - http://bmjopen.bmj.com/site/about/guidelines.xhtml

minutes to complete the HSS-40[21], which may limit its application, especially in extensive surveys. Though there exist shortened versions covering 25[21] and 32[22] items of the HIV stigma scale, the 12-item HIV stigma scale (HSS-12)[13] version of the Berger HIV stigma scale was examined in the present study as it facilitates the inclusion of HIV stigma in more extensive surveys. Furthermore, it has comparable psychometric properties to the full-length scale.[13] While evidence from other parts of the world[13] indicates that the HSS-12 is psychometrically sound, we are unaware of any study that has reported this scales' psychometric properties in the SSA context.

Empirical evidence indicates that sociodemographic characteristics such as age,[23 24] gender,[24-26] employment,[27] educational attainment,[28-30], and marital status,[31] are significantly correlated with HIV related stigma. However, the directionality is inconsistent. An explanation for the different findings regarding correlates and predictors of HIV related stigma might be due to the diverse research strategies applied and the sample composition. Research shows that stigma and disclosure of HIV status are interrelated phenomena for people living with HIV/AIDS.[32] Furthermore, persons who have not disclosed their HIV status exhibit higher levels of perceived HIV-related stigma and greater levels of concern about HIV disclosure.[33]

Despite the abundance of published reports on HIV related stigma and its predictors amongst specific sub-groups of the adult population, there is a paucity of research findings focusing on predictors of HIV related stigma across the entire adult population. Further, no study in the SSA context has tested for the validity and reliability of the HSS-12. This study aims to determine the correlates of HIV-related stigma among adults living with HIV from Kilifi, Coastal Kenya. Specifically, the study aims to: i) examine the psychometric properties of the 12-item Berger Stigma Scale; and ii) establish the correlates of stigma among adults living with HIV in Kilifi.

Methods

Study setting

This cross-sectional study was conducted at the Centre for Geographic Medicine Research-Coast, Kenya Medical Research Institute-Wellcome Trust Research Programme (KEMRI/WTRP). It was based at the Comprehensive Care and Research Centre (CCRC) in the Kilifi County Hospital (KCH). The majority of Kilifi County residents are poor (71.4% live below the poverty line), lack formal education, and earn a living mainly through subsistence

For peer review only - http://bmjopen.bmj.com/site/about/guidelines.xhtml

farming or fishing.[34-37] HIV prevalence in adults is estimated to be at 4.5%.[38] The CCRC offers clinical services such as management of opportunistic infections, HIV testing and counselling, family planning, nutritional counselling, cervical cancer screening, and serves as a research facility. About 60 patients are seen daily. By 2020, the clinic has enrolled over 9,000 patients of all ages.

Study participants

This data is part of a larger project focusing on diverse outcomes in adults living with HIV, including mental health and health-related quality of life. A cross-sectional survey of 450 study participants among patients attending an HIV care and treatment clinic at Kilifi County Hospital was conducted between February and April 2018 (Figure 1). The participation criteria were age (18-60 years old) with confirmed HIV positive status, on combination antiretroviral therapy, and informed consent to participate. Participants with an acute medical illness or cognitive difficulties at the time of enrolment/administration of questionnaire or could not understand and/or communicate in the national language (Kiswahili), which was used during the administration of all study instruments, were excluded. A research team member introduced the study to eligible participants when they visited the clinic for scheduled appointments. Those who consented to take part responded to the instruments at the clinic.

Data Collection Procedures

Study data were collected and managed using REDCap electronic data capture tools hosted at KEMRI Wellcome Trust Programme. Data collection instruments were interviewer-administered via android tablets, in the same order, and under the same administration environment. Research assistants underwent a 4-day training in research ethics and proper interviewing techniques (with role-plays) and were familiarised with the tablet-based questionnaires. The questionnaire administration took place in a quiet and private room within the CCRC in KCH, and the interview session lasted between 30 to 45 minutes.

Measures

HIV-related stigma: The short version (HSS-12) of the Berger HIV stigma scale[13] was used to assess patient-perceived HIV-related stigma under four dimensions: i) *personalised stigma*; ii) *disclosure concerns*; iii) *negative self-image;* and iv) *concerns with public attitudes,* each comprising a sub-scale of the instrument. *Personalised stigma* has been suggested to represent

For peer review only - http://bmjopen.bmj.com/site/about/guidelines.xhtml

the enacted stigma mechanism, *disclosure concerns,* and *concerns with public attitudes* dimensions have been proposed to represent anticipated stigma mechanism, and *negative self-image* has been proposed to represent internalised stigma mechanism.[11] Items on this scale are rated from 1-4, with (1) being "strongly disagree" and (4) "strongly agree." The possible score for each item ranges from 1 to 4 (3–12 for sub-scale), and a total score ranges between 12 and 48 and is derived from the summation of item scores. Higher scores designate a greater level of perceived HIV-related stigma.

Patient Health Questionnaire version 9 (PHQ-9) [39] was administered as a measure of depressive symptoms. The PHQ-9 is a nine-item scale rated on a Likert-type scale ranging from 0 "not at all" to 3 "nearly every day." Item scores are summated to derive a total score ranging from 0 to 27. It has previously been found to have good internal consistency (Cronbach alpha 0.78) and acceptable test-retest reliability (intraclass correlation coefficient [ICC]=0.59) when used among adults living with HIV infection in Kenya[40].

Generalised Anxiety Disorder (GAD-7)[41] was administered as a clinical measure for assessing generalised anxiety disorder based on DSM-IV criteria. The GAD-7 is a seven-item self-report instrument rated on a Likert-type scale ranging from 0 "not at all" to 3 "nearly every day." The scale score ranges from 0 to 21. There is reported evidence in support of the reliability and validity of this scale in Kenya.[42] Scores from PHQ-9 and GAD-7 were combined to generate a variable called CMD comorbidity, indicating the co-occurrence of depressive and anxiety symptoms.

Sociodemographic and asset index items: A sociodemographic questionnaire was used to collect information on the participants' age, gender, relationship status, educational level, employment status, and whom they currently shared a residence. Furthermore, an asset index previously used in this setting[43] was used to collect information about participants' socio-economic status (SES) based on disposable assets owned. Participants were asked for ownership of disposable items such as radio, television, refrigerator, gas, bicycle, motorcycle, and car. The final SES score had seven (7) items. A total asset score is calculated, and higher scores indicate a better SES. An asset index to estimate family wealth has been recommended as an alternative approach to estimating SES in settings where reliable data on family income may not be available.[44]

Clinical information: Participants' data were extracted from the clinic's medical record database and filled into a clinical record form. This information included participants' dates of

For peer review only - http://bmjopen.bmj.com/site/about/guidelines.xhtml

HIV-diagnosis, combination antiretroviral therapy initiation, most current combination antiretroviral therapy regimen, cluster of differentiation 4 (CD4) cell count, viral load, recent height and weight (for Body Mass Index (BMI) calculation), and data on World Health Organization (WHO) clinical staging. Participants' clinical information was retrieved from their clinical records after consent was granted. Patient-unique clinic numbers were used to access participants' medical records.

Instrument translation and cross-cultural adaptation

The English version of the HSS-12 was forward translated by two independent bilingual translators to Kiswahili and back-translated into English by two independent back translators (oblivious of the original version). A group of Kenyan HIV researchers bilingual and fluent in both Kiswahili and English and the translators had a harmonisation meeting to review the content, conceptual, semantic, and idiomatic equivalence of the questionnaires to ensure the cultural relevance of the HSS-12. The final version was obtained after the incorporation of changes emerging from pretesting.

Patient and public involvement

Patients were not involved in the design and conduct of this study.

Statistical analyses

Factor structure and measurement invariance across age-groups and gender

First, Confirmatory Factor Analysis (CFA) was used to examine the HIV-stigma scale's factor structure. A CFA model representing the Swahili version of the HSS-12 was set up and analysed with weighted least square mean and variance adjusted (WLSMV) using the lavaan[45] package in R statistical software[46] on all the 450 observations. The Goodness of fit was assessed using χ^2 test, Comparative Fit Index (CFI), Tucker Lewis Index (TLI), and root mean square error of approximation (RMSEA). The data was expected to have a good fit to the model if the χ^2 test was non-significant, CFI and TLI values were greater than 0.90, and RMSEA score was lower than 0.05.[47]

Secondly, after defining the model, Multi-Group Confirmatory Factor Analysis (MGCFA)[48] was used to test for measurement invariance of the HSS-12 for gender and age groups. Change in CFI (ΔCFI) has been suggested as a robust statistic for testing between-group invariance of

For peer review only - http://bmjopen.bmj.com/site/about/guidelines.xhtml

CFA models. Additionally, it has been recommended that invariance can be assumed when ΔCFI is ≤0.01 in absolute values.[49]

Internal construct validity and convergent validity

Means and standard deviations were used to evaluate the distribution of scores within the subscale and among the items. Itemised means and standard deviations were expected to be almost the same within the subscale, justifying item scores' aggregation into subscale scores.[50] The item-total correlation was used to evaluate internal construct validity. Each items' corrected item-total correlation coefficients were calculated and expected to exceed 0.4 and vary in range. Convergent validity was assessed using the Pearson correlation coefficient between HSS-12, PHQ-9, and GAD-7 scores. Correlation coefficients were interpreted as small (0.10–0.29), moderate (0.30–0.49), and large (0.49 and above).[51]

Reliability

Cronbach's alpha (α) was used to examine each subscale's internal consistency and overall scores of the Swahili version of the HSS-12. Cronbach's alpha was considered acceptable if greater than (>0.7).[52] The intra-class correlation coefficient (ICC) was used to examine test-retest of the Swahili version of the HSS-12 by correlating scores taken at two different time points (2 weeks apart) using the same measure administered to the same participant. ICC of 0.60 was considered marginal, 0.70 acceptable, and anything over 0.80 considered high.[53]

Sample characteristics and correlates

Frequencies and means (with percentages and standard deviations) were used to describe sample characteristics. Univariate and multivariable linear regression were used to assess factors associated with both stigma subscales and the overall stigma scale. In the regression model, stigma scores were expressed as a continuous measure. Independent variables included age, gender, marital status, education level, employment status, socioeconomic status (SES), body mass index (BMI), viral load, WHO clinical stages, months since HIV diagnosis, months since cART initiation, HIV status disclosure, self-reported opportunistic infections, and the co-occurrence of depressive and anxiety symptoms. Our review of the literature informed factors included in the model. All variables with $p<0.20$ were included in the multivariable regression model apart from viral load because participants had missing values (n=145). The final multivariable models were generated using a backward stepwise approach by eliminating all variables independently with $p>0.05$. Assumptions of linear regression testing were visually inspected through histograms (linearity), normal probability plots (normality), and plots of

For peer review only - http://bmjopen.bmj.com/site/about/guidelines.xhtml

residual versus predicted values (homoscedasticity). Multicollinearity was assessed using the variance inflation factor (VIF). There were no multicollinearity problems. Modelling was undertaken five times in total: once to predict overall stigma and once to predict each of the four subscales. R (version 3.6.3) statistical software package was used to explore the construct validity of the HSS-12. All other analyses were run using (Stata version 15.0) statistical software package.

Results

Sample Characteristics

The 450 participants had a median age of 43 years (IQR = 36-50), ranging from 18 to 60 years. The vast majority of the sample were female (79.1%), had attained basic primary level education (53.1%), lived with a family member (82.4%), and were unemployed (59.8%). Less than half of the study participants (43.8%) were either separated, divorced, or widowed. The mean BMI was within the normal range (mean [SD] = 22.4 [4.8]). Most study participants had disclosed their HIV status to others (94.0%). The median time since HIV diagnosis was 8.8 years (IQR = 4.67-11.50), ranging from 0 to 18 years. 417(93.7%) were in stage 1 of the WHO clinical staging and 425 (95.3%) on the first-line cART regimen (Table 1). The median time elapsed since cART initiation was 6.7 years (IQR = 3.67-10.00). At the time of the interview, less than a fifth (18.4%) of the study participants had an opportunistic infection.

Perceived overall stigma scores ranged from 12 to 48, with a median score of 28 (IQR = 23-33). Using PHQ-9 and GAD-7 cut-off score of ≥10, which have been shown to maximise specificity and sensitivity for depression[54] and general anxiety disorder[41] screening, the overall prevalence of depressive and anxiety was 13.8% and 5.3%, respectively, among enrolled participants. The co-occurrence of depressive and anxiety symptoms was present in 4.7% of the study participants.

Factor structure and measurement invariance across age groups and gender

Supplementary **Error! Reference source not found.** presents CFA results with standardised correlation coefficients. Our hypothesised model that the overall stigma scale comprises four sub-scales correlated was confirmed given the observed fit indexes. The χ^2 test was statistically significant (χ^2 = 91.982, df= 50, p=0.000) but alternate fit measures indicated acceptable fit; RMSEA: 0.044; CFI:0.966 and TLI: 0.955. These results generally indicate that the data had a good fit to the model and that we can confidently use both total and sub-scale scores in this

For peer review only - http://bmjopen.bmj.com/site/about/guidelines.xhtml

population. Measurement invariance across age groups and gender was entirely supported because ΔCFIs are lower than 0.01 in all models suggesting that measurement invariance can be assumed.

Internal construct validity and convergent validity

The factor loading of all items on the hypothesised scale was good except for item 6 (0.21) under the disclosure concern subscale. Convergent validity of the HSS-12 was demonstrated by the small to moderate correlations between HSS-12 and the correlation with the following relevant measures: GAD-7 ($r = 0.368$, $p<0.001$) and PHQ-9 ($r = 0.328$, $p<0.001$) Table 2.

Reliability: Internal consistency and test-retest

Cronbach's α for the subscales and overall scale were all >0.7 (see Table 2) except for the disclosure concern sub-scale, which was 0.53 (95%CI: 0.46, 0.60). The test-retest reliability of the short 12-item version of the HIV stigma scale was excellent, 0.92 (95%CI: 0.87, 0.95). Additionally, Table 2 presents descriptive statistics for the stigma scale on the item level and subscale level. Corrected item-total correlation coefficients were >0.4 for all the items apart from one item (0.21) in the disclosure concerns subscale. There is a variation in the range of 0.46-0.88, indicating that the intended stigma concepts' broadness had been captured.

Correlates of perceived HIV related stigma

In the univariate model, it was found that being female, being separated, divorced or widowed, having primary or no level of education, being self-employed or unemployed, having a low asset index score, having a viral load of >1000 copies/ml, decreased duration since HIV diagnosis, decreased duration since cART initiation, HIV status non-disclosure, having any current opportunistic infection and co-occurrence of depression and anxiety symptoms were significantly associated with overall HIV stigma scores.

Personalised stigma was significantly associated with being female, being single or either separated, divorced or widowed, self-employed or unemployed, having a low asset index score, having a viral load of >1000 copies/ml, having any current opportunistic infection, and the co-occurrence of depressive and anxiety symptoms. *Disclosure concern* was significantly associated with either being separated, divorced or widowed, having no level of education, having a low asset index score, less time elapsed since HIV diagnosis, less time elapsed since cART initiation, and HIV status non-disclosure. *Concern with public attitudes* was significantly associated with being female, having primary or no level of education, decreased

For peer review only - http://bmjopen.bmj.com/site/about/guidelines.xhtml

duration since cART initiation, and the co-occurrence of depressive and anxiety symptoms. *Negative self-image* was significantly associated with either being separated, widowed or divorced, having no level of education, being self-employed or unemployed, having a viral load of >1000 copies/ml, decreased duration since HIV diagnosis, decreased duration since cART initiation, having any current opportunistic infection and the co-occurrence of depressive and anxiety symptoms (Table 3).

When a multiple linear regression model was run, it was found that being female (β=2.001, *95%CI: 0.21, 3.80,* p=0.029), HIV status disclosure (β=4.237, 95%CI: 1.27, 7.20, p=0.005) and co-occurrence of depressive and anxiety symptoms (β=6.670, *95%CI: 3.40, 9.94,* p<0.001) were significant predictors of perceived HIV stigma. Having no education was borderline statistically significant (β=3.318, *95%CI: -.01, 6.65,* p=0.051). Regression results indicated that the model explained 10.2% of the variance and that the model was a significant predictor of perceived HIV stigma F (6, 395) = 7.46, p<.001).

Concerning the four subscales, we found that *personalised stigma* was positively correlated with being female and the co-occurrence of depressive and anxiety symptoms. *Disclosure concern* was inversely correlated with duration since HIV diagnosis and positively correlated with having no level of education and HIV status non-disclosure. *Concerns with public attitudes* were positively correlated with being female. *Negative self-image* was positively correlated with having no level of education and the co-occurrence of depressive and anxiety symptoms (Table 4).

Discussion

This cross-sectional analysis of data from adults living with HIV observed that the HSS-12 presents excellent psychometric properties. Additionally, we observed that stigma was associated with both physical and mental well-being. According to our study, correlates of HIV related stigma include being female, HIV status non-disclosure, and the co-occurrence of depressive and anxiety symptoms. Furthermore, although having no education was borderline statistically significant, we would still suggest focusing on people with no education as a risk group from a programmatic point of view.

For peer review only - http://bmjopen.bmj.com/site/about/guidelines.xhtml

Factor structure, measurement invariance, validity and reliability of the short 12-item Swahili version of the HIV Stigma Scale

The study examined the stigma scale's psychometric properties to assess its usefulness and describe the correlates of HIV-related stigma among adults living with HIV in Kilifi. Reliability and validity were acceptable, and confirmatory factor analysis supported the four-factor solution measuring the four dimensions of HIV stigma. Cronbach's alpha for the HSS-12 among the Kenyan population is similar to the Swedish population in which the scale was developed.[13] Although Cronbach's alpha for the adapted HSS-12 sub-scales was slightly lower (0.53-0.84) than the initial version of HSS-12 (0.80-0.88), its' alpha for the total scale was 0.80 suggesting good internal consistency.

Measurement invariance of the Swahili HSS-12 was evaluated and confirmed across main interest groups: gender and age. Our results indicated that the measurement model of the Swahili HSS-12 as a patient-reported outcome to measure perceived HIV stigma among adults is comparable across age groups and gender (Table 5).

Test-retest reliability, an indicator of scale stability over time, was of acceptable levels. The original HSS-40 has been used in diverse settings[12 55] among adults 18 years and above reporting a test re-test reliability between (ICC=0.89-0.92). To the best of our knowledge, no study has reported the test re-test reliability of the HSS-12.

We examined the construct validity of the scale using CFA since its hypothesised structure has been published. Our results indicated that the hypothesised model fit the data well and was almost similar to what was reported by a study conducted in Sweden[13]. These results indicate that one can use both the total scores and the subscale scores and interpret the results in confidence, knowing that the items fit well together. HSS-12 evidenced convergent validity by being correlated with PHQ-9, a measure of depression, and GAD-7, a measure of anxiety in conventional ways.

The HSS-12 was reliable and valid for detecting stigma among adults living with HIV at the Kenyan Coast. Consequently, HSS-12 can be practically used as a brief screening tool for stigma-related problems both for research and clinical purposes. Future research could examine its predictive validity and evaluate its sensitivity to changes. This information would be crucial in determining its usefulness as an evaluation tool for programmes and interventions.

For peer review only - http://bmjopen.bmj.com/site/about/guidelines.xhtml

Correlates of Stigma

Being female was positively associated with increased perceived HIV-related stigma scores, *personalised stigma,* and *concern with public attitudes*. This finding agrees with previous studies from SSA[56] and outside[57,27] that reported a positive association between female gender and perceived HIV related stigma. Research shows that females are more likely to suffer from stigma in patriarchal societies like ours than males.[58 59] Research has established that the African society is less tolerant of HIV infected females than it is of HIV infected men.[60 61] Due to women's subordinate status in society, they are often stigmatised as vectors of transmission.[62] Furthermore, the common belief that HIV is caused by indecent sexual behaviour has worse societal consequences for women who are expected to be monogamous, unlike men in most African societies.[60] Women are often blamed counterfactually to be responsible for HIV transmission.[60] Similar processes can be assumed to be at work in the Kenyan coastal region.

HIV status disclosure was positively associated with overall HIV related stigma scores and *disclosure concerns* with persons who had not disclosed their HIV status reporting greater levels of concern about HIV disclosure concerns. Anakwa and colleagues found that PLWHA with higher levels of perceived HIV-related stigma reported greater levels of HIV disclosure concerns, therefore, less likely to disclose their status.[33] From our study, only 6% had not disclosed their status to anyone. HIV status non-disclosure might be a protective behaviour for a PLWHA to conceal their status, evade adverse reactions towards themselves, weigh other people's reactions, and as a sign of concern about the implication of their disclosure on their disclosure targets.[63 64] Further, disclosure is not only about how or whom to disclose to, but it also entails finding good opportunities to disclose or devise means of keeping ones' status and/or medication a secret to enhance access and adherence to their treatment regimen.

The co-occurrence of depressive and anxiety symptoms influenced overall HIV related stigma scores, *personalised stigma,* and *negative self-image*. This finding corroborates previous studies among PLWHA carried out within SSA[29 65, 25], and outside[66 67], which have invariably found a significant association between HIV-related stigma and depressive symptoms. Liu and colleagues[68] reported that the more stigma PLWHA perceived, the more anxiety they experienced. Similarly, we report that HIV related stigma is significantly associated with the co-occurrence of depressive and anxiety symptoms. Additionally, an individual's perception of themselves in light of their diagnosis appears to trigger depression.[69] Screening for

For peer review only - http://bmjopen.bmj.com/site/about/guidelines.xhtml

depression, anxiety, and HIV-related stigma might provide insights on interventions that may promote a positive attitude and positive self-image, thereby reducing depression, anxiety, and stigma, leading to their psychological and physical well-being. Given the cross-sectional nature of the study, we cannot claim causality. However, the association between co-occurrence of depressive and anxiety symptoms and stigma provides the impetus for: a) longitudinal studies to elucidate causal pathways; and b) targeted interventions to address both stigma and mental health to improve health outcomes of adults living with HIV.

Other factors influencing the four subscales were also established. Having no level of education was positively associated with higher reported *disclosure concerns* and *negative self-image,* corroborating findings of studies carried out in Nigeria[70] and the USA.[71] Lower levels of education may lead to less exposure, lack of or little knowledge about HIV infection and transmission. In contrast, higher levels of education might lead to higher levels of knowledge, providing exposure to new ways of thinking and new sources of information about the HIV pandemic resulting in the reduction of less supportive attitudes towards PLWHA.[72 73] Previous research has demonstrated that people with high levels of knowledge of the transmission routes for HIV consistently had more supportive attitudes towards those with HIV demonstrating the role that knowledge has in reducing the misconceptions that act to create fear and shape stigma.[72]

Months since HIV diagnosis was inversely associated with *disclosure concerns,* with persons with a more recent diagnosis reporting greater levels of concern about HIV status disclosure. This is consistent with a study among people living with HIV/AIDS (PLWHA) in China[74] and among African Americans.[71] This finding suggests that living longer with HIV is associated with positive outcomes because PLWHA are likely to adjust over time to their HIV positive status, receive more information, develop greater insights and understanding of the disease and establish psychological mechanisms to better cope with HIV stigma leading to lower levels of perceived HIV stigma.

Strengths and limitations of this study

We recognise several potential limitations in this study. First, the study was in a clinical setting where our study sample consisted of adults living with HIV on cART. Compared to untreated individuals living with HIV, it is likely that levels of HIV stigma would be lower in our sample because it has been shown that access to ART lowers stigma.[75-77] Secondly, this study is cross-sectional, and hence, causality for the observed significant associations cannot be inferred. We

For peer review only - http://bmjopen.bmj.com/site/about/guidelines.xhtml

can also not conclude how individuals may experience stigma over time because of the study design limitation. Finally, findings may not be generalisable to all adults living with HIV in Kenya as data were collected from one geographical setting and excluded adults older than 60 years.

Conclusions and implications

From the study, the 12-item short version of the Berger HIV stigma scale[13] had good psychometric properties and can be recommended for research purposes. The current study suggests that women, those who have not disclosed, and those experiencing co-occurring symptoms of depressive and anxiety symptoms, experience a higher level of perceived HIV stigma in Coastal Kenya. This finding is useful in designing future interventions to improve the quality of life of people living with HIV/AIDS. We propose interventions need to take into account gender to address the specific needs of women, people who have not disclosed their HIV status, and those exhibiting symptoms of depression and anxiety, thereby improving their quality of life. Additionally, it would be prudent to design interventions that focus on people with no education as a risk group who would experience high levels of HIV perceived stigma from a programmatic perspective. All these interventions will help in bettering both the physical and mental well-being of HIV-infected adults.

Acknowledgements

We thank our participants for taking the time and effort to participate in this study and the Comprehensive Care and Research Centre (CCRC) staff in the Kilifi County Hospital HIV care and treatment clinic for their unrelenting support. We appreciate Patrick Katana Vidzo for helping with the entry of clinical data. The authors acknowledge the Kenya Medical Research Institute (KEMRI) Director for permission to publish this work.

Footnotes

Author Contributions: SWW, CN, and AA conceptualised the study. SWW, MKN and AA designed the study. PM formulated study questions for tablet administration and managed the data. SWW and MKN supervised data collection. SWW, MKN, and AM participated in data collection. SWW and MKN analysed the data. SWW, MKN, PM, AM, SL, CN and AA contributed to interpreting the data. SWW wrote the first draft of the manuscript. All authors reviewed subsequent versions of the manuscript and approved the final version for submission.

For peer review only - http://bmjopen.bmj.com/site/about/guidelines.xhtml

The corresponding author affirms that all listed authors meet authorship criteria and that no other author meeting the criteria has been omitted.

Funding: This work was supported by funding from the Medical Research Council (Grant number MR/M025454/1) to AAA. This award is jointly funded by the UK Medical Research Council (MRC) and the UK Department for International Development (DFID) under the MRC/DFID concordant agreement and is also part of the EDCTP2 program supported by the European Union.

Disclaimer: The funders had no role in the design, collection, analysis, interpretation and manuscript writing.

Competing interests: The authors wish to declare no potential conflict of interest.

Ethical approval: The local institutional review board, Scientific and Ethics Review Board (SERU; Ref KEMRI/SERU/CGMR-C/108/3594), granted ethical approval to recruit participants into the study. We obtained authorisation to work in the HIV care and treatment clinic from the Ministry of Health, County government of Kilifi (Ref HP/KCHS/VOL.VIX/65). Study participants provided written, informed consent to be part of the study.

Transparency: The lead author (SWW) confirms that the manuscript is an honest, accurate, and sincere account of the research being reported; no important aspects of the research have been omitted; and that explanations for any discrepancies from the research as planned (and, if relevant, registered) have been provided.

Data sharing statement: No additional data are available. Anyone interested in accessing the data reported in this article is free to write to the Data Governance Committee of the KEMRI Wellcome Trust Research Programme, review the application and advise as appropriate, and ensure that uses are compatible with the consent obtained from participants for data collection. Requests can be sent to the coordinator of the Data Governance Committee using the following email: dgc@kemri-wellcome.org.

For peer review only - http://bmjopen.bmj.com/site/about/guidelines.xhtml

REFERENCES

1. UNAIDS. Global HIV & AIDS statistics — 2020 fact sheet [Available from: https://www.unaids.org/en/resources/fact-sheet

files/3352/fact-sheet.html.
2. NACC. Kenya AIDS Response Progress Report 2014: Progress towards Zero. Nairobi, Kenya: National AIDS Control Council of Kenya, 2014.
3. Goffman E. Stigma. Notes on the Management of Spoiled Identity. New York: Simon and Shuster: Inc, 1963.
4. Jones EE, Farina A, Hastorf AH, et al. Social stigma: the psychology of marked relationships. New York: W.H. Freeman 1984.
5. Maluccio JA, Wu F, Rokon RB, et al. Assessing the Impact of Food Assistance on Stigma Among People Living with HIV in Uganda Using the HIV/AIDS Stigma Instrument-PLWA (HASI-P). *AIDS and Behavior* 2017;21(3):766-82. doi: 10.1007/s10461-016-1476-9 [published Online First: 2016/07/04]
6. Dahlui M, Azahar N, Bulgiba A, et al. HIV/AIDS Related Stigma and Discrimination against PLWHA in Nigerian Population. *PLoS One* 2015;10(12):e0143749. doi: 10.1371/journal.pone.0143749 [published Online First: 2015/12/15]
7. Toth S, J AY, DePinto N. HIV stigma: perceptions from HIV-positive and HIV-negative patients in a community dental clinic. *J Dent Res Dent Clin Dent Prospects* 2016;10(4):263-69. doi: 10.15171/joddd.2016.042 [published Online First: 2017/01/18]
8. Nyblade L, Stangl A, Weiss E, et al. Combating HIV stigma in health care settings: what works? *J Int AIDS Soc* 2009;12:15. doi: 10.1186/1758-2652-12-15 [published Online First: 2009/08/08]
9. Clark HJ, Lindner G, Armistead L, et al. Stigma, disclosure, and psychological functioning among HIV-infected and non-infected African-American women. *Women & health* 2004;38(4):57-71.
10. Lindberg MH, Wettergren L, Wiklander M, et al. Psychometric evaluation of the HIV stigma scale in a Swedish context. *PLoS One* 2014;9(12):e114867. doi: 10.1371/journal.pone.0114867 [published Online First: 2014/12/19]
11. Earnshaw VA, Chaudoir SR. From conceptualizing to measuring HIV stigma: a review of HIV stigma mechanism measures. *AIDS Behav* 2009;13(6):1160-77. doi: 10.1007/s10461-009-9593-3 [published Online First: 2009/07/29]
12. Berger BE, Ferrans CE, Lashley FR. Measuring stigma in people with HIV: Psychometric assessment of the HIV stigma scale¶. *Research in nursing & health* 2001;24(6):518-29.
13. Reinius M, Wettergren L, Wiklander M, et al. Development of a 12-item short version of the HIV stigma scale. *Health and Quality of Life Outcomes* 2017;15(1):115. doi: 10.1186/s12955-017-0691-z [published Online First: 2017/06/01]
14. Kingori C, Reece M, Obeng S, et al. Psychometric evaluation of a cross-culturally adapted felt stigma questionnaire among people living with HIV in Kenya. *AIDS Patient Care STDS* 2013;27(8):481-8. doi: 10.1089/apc.2012.0403 [published Online First: 2013/08/24]
15. Wiklander M, Rydstrom LL, Ygge BM, et al. Psychometric properties of a short version of the HIV stigma scale, adapted for children with HIV infection. *Health Qual Life Outcomes* 2013;11(1):195. doi: 10.1186/1477-7525-11-195 [published Online First: 2013/11/15]
16. Holzemer WL, Uys LR, Chirwa ML, et al. Validation of the HIV/AIDS Stigma Instrument—PLWA (HASI-P). 2007;19(8):1002-12.

For peer review only - http://bmjopen.bmj.com/site/about/guidelines.xhtml

17. Stigma among mothers with HIV/AIDS. XIII International AIDS Conference, Durban, South Africa; 2000.
18. Fife BL, Wright ER. The dimensionality of stigma: a comparison of its impact on the self of persons with HIV/AIDS and cancer. *J Health Soc Behav* 2000;41(1):50-67. [published Online First: 2000/04/06]
19. Herek GM, Capitanio JP. Public reactions to AIDS in the United States: a second decade of stigma. *Am J Public Health* 1993;83(4):574-7. doi: 10.2105/ajph.83.4.574 [published Online First: 1993/04/01]
20. Sowell RL, Seals BF, Moneyham L, et al. Quality of life in HIV-infected women in the south-eastern United States. *AIDS Care* 1997;9(5):501-12. doi: 10.1080/713613191 [published Online First: 1997/12/24]
21. Jeyaseelan L, Kumar S, Mohanraj R, et al. Assessing HIV/AIDS stigma in South India: validation and abridgement of the Berger HIV stigma scale. 2013;17(1):434-43.
22. Bunn JY, Solomon SE, Miller C, et al. Measurement of stigma in people with HIV: a reexamination of the HIV Stigma Scale. *AIDS Educ Prev* 2007;19(3):198-208. doi: 10.1521/aeap.2007.19.3.198 [published Online First: 2007/06/15]
23. Emlet CA, Brennan DJ, Brennenstuhl S, et al. The impact of HIV-related stigma on older and younger adults living with HIV disease: does age matter? *AIDS care* 2015;27(4):520-28.
24. Pannetier J, Lelievre E, Le Coeur S. HIV-related stigma experiences: Understanding gender disparities in Thailand. *AIDS Care* 2016;28(2):170-8. doi: 10.1080/09540121.2015.1096888 [published Online First: 2015/10/20]
25. Endeshaw M, Walson J, Rawlins S, et al. Stigma in Ethiopia: association with depressive symptoms in people with HIV. *AIDS Care* 2014;26(8):935-9. doi: 10.1080/09540121.2013.869537 [published Online First: 2014/01/03]
26. Datta S, Bhattacherjee S, Sherpa PL, et al. Perceived HIV Related Stigma among Patients Attending ART Center of a Tertiary Care Center in Rural West Bengal, India. *Journal of clinical and diagnostic research : JCDR* 2016;10(10):VC09-VC12. doi: 10.7860/JCDR/2016/21366.8669 [published Online First: 2016/11/29]
27. Sorsdahl KR, Mall S, Stein DJ, et al. The prevalence and predictors of stigma amongst people living with HIV/AIDS in the Western Province. *AIDS Care* 2011;23(6):680-5. doi: 10.1080/09540121.2010.525621 [published Online First: 2011/03/02]
28. Yeo TED, Chu TH. Social-cultural factors of HIV-related stigma among the Chinese general population in Hong Kong. *AIDS Care* 2017;29(10):1255-59. doi: 10.1080/09540121.2017.1282601 [published Online First: 2017/01/24]
29. Cuca YP, Onono M, Bukusi E, et al. Factors associated with pregnant women's anticipations and experiences of HIV-related stigma in rural Kenya. *AIDS Care* 2012;24(9):1173-80. doi: 10.1080/09540121.2012.699669 [published Online First: 2012/07/18]
30. Girma E, Gebretsadik LA, Kaufman MR, et al. Stigma against people with HIV/AIDS in rural Ethiopia, 2005 to 2011: signs and predictors of improvement. *AIDS Behav* 2014;18(6):1046-53. doi: 10.1007/s10461-013-0611-0 [published Online First: 2013/09/28]
31. Takada S, Weiser SD, Kumbakumba E, et al. The Dynamic Relationship Between Social Support and HIV-Related Stigma in Rural Uganda. *Annals of Behavioral Medicine* 2014;48(1):26-37. doi: 10.1007/s12160-013-9576-5
32. Emlet CA. A comparison of HIV stigma and disclosure patterns between older and younger adults living with HIV/AIDS. *Aids Patient Care and Stds* 2006;20(5):350-58. doi: DOI 10.1089/apc.2006.20.350

For peer review only - http://bmjopen.bmj.com/site/about/guidelines.xhtml

33. Anakwa NO, Teye-Kwadjo E, Kretchy IA. Effect of HIV-Related Stigma and HIV-Related Stress on HIV Disclosure Concerns: a Study of HIV-Positive Persons on Antiretroviral Therapy at Two Urban Hospitals in Ghana. *Applied Research in Quality of Life* 2020:1-16.
34. Scott JA, Bauni E, Moisi JC, et al. Profile: The Kilifi Health and Demographic Surveillance System (KHDSS). *Int J Epidemiol* 2012;41(3):650-7. doi: 10.1093/ije/dys062 [published Online First: 2012/05/01]
35. Allocation(CRA) CoR. Kenya County Fact sheets: ; 2011 [Available from: http://siteresources.worldbank.org/INTAFRICA/Resources/257994-1335471959878/Kenya_County_Fact_Sheets_Dec2011.pdf accessed 22nd August 2018.
36. Statistics KNBo, Africa SfIDE. Exploring Kenya's Inequality: Pulling Apart or Pooling Together?: KNBS and SIDS Nairobi, 2013.
37. Ngugi E, Kipruto S, Samoei PJKNBoS, et al. Exploring Kenya's Inequality: Pulling Apart or Pooling Together? Kilifi County. 2013
38. NACC. Kenya HIV County Profiles - 2016: National AIDS Control Council; 2016 [49-52]. Available from: http://nacc.or.ke/wp-content/uploads/2016/12/Kenya-HIV-County-Profiles-2016.pdf accessed 20th August 2018.
39. Spitzer RL, Kroenke K, Williams JB. Validation and utility of a self-report version of PRIME-MD: the PHQ primary care study. Primary Care Evaluation of Mental Disorders. Patient Health Questionnaire. *JAMA* 1999;282(18):1737-44. doi: 10.1001/jama.282.18.1737 [published Online First: 1999/11/24]
40. Monahan PO, Shacham E, Reece M, et al. Validity/reliability of PHQ-9 and PHQ-2 depression scales among adults living with HIV/AIDS in western Kenya. *J Gen Intern Med* 2009;24(2):189-97. doi: 10.1007/s11606-008-0846-z [published Online First: 2008/11/26]
41. Spitzer RL, Kroenke K, Williams JB, et al. A brief measure for assessing generalized anxiety disorder: the GAD-7. *Arch Intern Med* 2006;166(10):1092-7. doi: 10.1001/archinte.166.10.1092 [published Online First: 2006/05/24]
42. Osborn TL, Venturo-Conerly KE, Wasil AR, et al. Depression and anxiety symptoms, social support, and demographic factors among Kenyan high school students. *Journal of Child Family Studies* 2019:1-12.
43. Nyongesa MK, Mwangala PN, Mwangi P, et al. Neurocognitive and mental health outcomes and association with quality of life among adults living with HIV: a cross-sectional focus on a low-literacy population from coastal Kenya. *BMJ Open* 2018;8(9):e023914. doi: 10.1136/bmjopen-2018-023914 [published Online First: 2018/09/19]
44. Filmer D, Pritchett LHJD. Estimating wealth effects without expenditure data—or tears: an application to educational enrollments in states of India. *%J Demography* 2001;38(1):115-32.
45. Rosseel Y. Lavaan: An R package for structural equation modeling and more. Version 0.5–12 (BETA). *Journal of statistical software* 2012;48(2):1-36.
46. RCore T. R: A language and environment for statistical computing. R Foundation for Statistical Computing, Vienna, Austria. *URL http://www R-project org* 2016
47. Blunch N. Introduction to structural equation modeling using IBM SPSS statistics and AMOS: Sage 2012.
48. Van de Schoot R, Lugtig P, Hox J. A checklist for testing measurement invariance. *European Journal of Developmental Psychology* 2012;9(4):486-92.

For peer review only - http://bmjopen.bmj.com/site/about/guidelines.xhtml

49. Cheung GW, Rensvold RB. Assessing extreme and acquiescence response sets in cross-cultural research using structural equations modeling. *Journal of cross-cultural psychology* 2000;31(2):187-212.
50. Ware Jr JE, Gandek BJJoce. Methods for testing data quality, scaling assumptions, and reliability: the IQOLA Project approach. 1998;51(11):945-52.
51. Cohen J. Statistical power analysis for the behavioural sciences, 2nd edn.(Hillsdale, NJ: L. Erlbaum Associates). 1988
52. Tabachnik B, Fidell L. Using multivariate statistics (6e éd.). Boston, É.-U. *U: Pearson* 2013
53. Streiner DL. A checklist for evaluating the usefulness of rating scales. *Canadian journal of psychiatry Revue canadienne de psychiatrie* 1993;38(2):140-8. doi: 10.1177/070674379303800214 [published Online First: 1993/03/01]
54. Akena D, Joska J, Obuku EA, et al. Sensitivity and specificity of clinician administered screening instruments in detecting depression among HIV-positive individuals in Uganda. *AIDS Care* 2013;25(10):1245-52. doi: 10.1080/09540121.2013.764385 [published Online First: 2013/02/13]
55. Jeyaseelan L, Kumar S, Mohanraj R, et al. Assessing HIV/AIDS stigma in south India: validation and abridgement of the Berger HIV Stigma scale. *AIDS Behav* 2013;17(1):434-43. doi: 10.1007/s10461-011-0128-3 [published Online First: 2012/01/17]
56. Nattabi B, Li J, Thompson SC, et al. Factors associated with perceived stigma among people living with HIV/AIDS in post-conflict northern Uganda. *AIDS Education and Prevention* 2011;23(3):193-205.
57. Swendeman D, Rotheram-Borus MJ, Comulada S, et al. Predictors of HIV-related stigma among young people living with HIV. *Health Psychol* 2006;25(4):501-9. doi: 10.1037/0278-6133.25.4.501 [published Online First: 2006/07/19]
58. Asiedu GB, Myers-Bowman KS. Gender differences in the experiences of HIV/AIDS-related stigma: a qualitative study in Ghana. *Health Care Women Int* 2014;35(7-9):703-27. doi: 10.1080/07399332.2014.895367 [published Online First: 2014/02/26]
59. Mugoya GC, Ernst K. Gender differences in HIV-related stigma in Kenya. *AIDS Care* 2014;26(2):206-13. doi: 10.1080/09540121.2013.808733 [published Online First: 2013/06/26]
60. Mbonu NC, van den Borne B, De Vries NK. Stigma of People with HIV/AIDS in Sub-Saharan Africa: A Literature Review. *J Trop Med* 2009;2009:145891. doi: 10.1155/2009/145891 [published Online First: 2009/01/01]
61. Amuyunzu-Nyamongo M, Okeng'o L, Wagura A, et al. Putting on a brave face: the experiences of women living with HIV and AIDS in informal settlements of Nairobi, Kenya. *AIDS Care* 2007;19 Suppl 1(sup1):S25-34. doi: 10.1080/09540120601114618 [published Online First: 2007/03/17]
62. Muyinda H, Seeley J, Pickering H, et al. Social aspects of AIDS-related stigma in rural Uganda. 1997;3(3):143-47.
63. Linda P. To tell or not to tell: negotiating disclosure for people living with HIV on antiretroviral treatment in a South African setting. *SAHARA J* 2013;10 Suppl 1(sup1):S17-27. doi: 10.1080/02664763.2012.755320 [published Online First: 2013/07/17]
64. Evangeli M, Wroe AL. HIV Disclosure Anxiety: A Systematic Review and Theoretical Synthesis. *AIDS Behav* 2017;21(1):1-11. doi: 10.1007/s10461-016-1453-3 [published Online First: 2016/07/14]
65. Nyongesa MK, Mwangi P, Wanjala SW, et al. Prevalence and correlates of depressive symptoms among adults living with HIV in rural Kilifi, Kenya. *BMC Psychiatry*

For peer review only - http://bmjopen.bmj.com/site/about/guidelines.xhtml

2019;19(1):333. doi: 10.1186/s12888-019-2339-5 [published Online First: 2019/11/05]
66. Charles B, Jeyaseelan L, Pandian AK, et al. Association between stigma, depression and quality of life of people living with HIV/AIDS (PLHA) in South India–a community based cross sectional study. 2012;12(1):463.
67. Yi S, Chhoun P, Suong S, et al. AIDS-related stigma and mental disorders among people living with HIV: a cross-sectional study in Cambodia. 2015;10(3):e0121461.
68. Liu Y, Gong H, Yang G, et al. Perceived stigma, mental health and unsafe sexual behaviors of people living with HIV/AIDS. 2014;39(7):658-63.
69. Onyebuchi-Iwudibia O, Brown A. HIV and depression in Eastern Nigeria: the role of HIV-related stigma. *AIDS Care* 2014;26(5):653-7. doi: 10.1080/09540121.2013.844761 [published Online First: 2013/10/19]
70. Oke OO, Akinboro AO, Olanrewaju FO, et al. Assessment of HIV-related stigma and determinants among people living with HIV/AIDS in Abeokuta, Nigeria: A cross-sectional study. *SAGE Open Medicine* 2019;7:2050312119869109.
71. Galvan FH, Davis EM, Banks D, et al. HIV stigma and social support among African Americans. 2008;22(5):423-36.
72. Stephenson R. Community factors shaping HIV-related stigma among young people in three African countries. *AIDS care* 2009;21(4):403-10.
73. Cianelli R, Villlegas N, De Oliveira G, et al. Predictors of HIV enacted stigma among Chilean women. 2015;24(17-18):2392-401.
74. Li Z, Morano JP, Khoshnood K, et al. HIV-related stigma among people living with HIV/AIDS in rural Central China. 2018;18(1):453.
75. Chan BT, Tsai AC, Siedner MJ. HIV Treatment Scale-Up and HIV-Related Stigma in Sub-Saharan Africa: A Longitudinal Cross-Country Analysis. *Am J Public Health* 2015;105(8):1581-7. doi: 10.2105/AJPH.2015.302716 [published Online First: 2015/06/13]
76. Wolfe WR, Weiser SD, Leiter K, et al. The impact of universal access to antiretroviral therapy on HIV stigma in Botswana. *Am J Public Health* 2008;98(10):1865-71. doi: 10.2105/AJPH.2007.122044 [published Online First: 2008/08/16]
77. Jurgensen M, Sandoy IF, Michelo C, et al. Effects of home-based voluntary counselling and testing on HIV-related stigma: findings from a cluster-randomized trial in Zambia. *Soc Sci Med* 2013;81:18-25. doi: 10.1016/j.socscimed.2013.01.011 [published Online First: 2013/02/21]

For peer review only - http://bmjopen.bmj.com/site/about/guidelines.xhtml

Table 1: Participant's sociodemographic characteristics

Sample Characteristics	Total sample N=450	%
**Sociodemographic characteristics**		
Age – years (18-60), Median (IQR)	43(14)	
Gender		
Female	356	79.1
Male	94	20.9
Marital Status		
Married/cohabiting	196	43.6
Separated /Divorced/Widowed	197	43.8
Single/Never Married	57	12.7
Education		
Tertiary	22	4.9
Secondary	66	14.7
Primary	239	53.1
None	123	27.3
Employment		
Formally employed	53	11.8
Self-employed	117	26.0
Other	11	2.4
Unemployed (including students)	269	59.8
Currently living with		
Family	371	82.4
Relative/friend	10	2.2
Alone	69	15.3
Asset index score [a] – mean (SD)	1.2(1.4)	1.4
Perceived HIV-stigma score [b] – mean (SD)	28.4(7.7)	7.7
Any current chronic illness		
No	413	91.8
Yes	37	8.2
**Clinical characteristics**		
BMI – kg/m2, mean (SD), OM = 4	22.4 (4.8)	
cART regimen, OM = 4		
First-line	425	95.3
Second line	21	4.7
Viral load, OM = 145		
≤ 1000 copies/mL	265	89.6
> 1000 copies/mL	40	13.1
WHO clinical stage, OM = 5		
Stage 1	417	93.7
Stage 2	22	4.9
Stage 3	3	0.7
Stage 4	3	0.7
Months since HIV diagnosis – Median (IQR)	106 (82)	
Months since cART initiation – Median (IQR)	80.5 (76)	
**Treatment Characteristics**		
HIV status disclosure		
Yes	423	94.0
No	27	6.0
Any current opportunistic infection		
No	367	81.6
Yes	83	18.4

Notes: OM = Observation with missing value, SD = Standard deviation, a - score range = 0 to 7, b – score range = 12 to 48, IQR = Interquartile range

For peer review only - http://bmjopen.bmj.com/site/about/guidelines.xhtml

Table 2: Descriptive statistics for items and subscales in the short form 12-item Swahili version of the HIV Stigma Scale

				Reliability		Validity				
						Convergent		Construct		
Item	Mean item score[a] (SD)	Corrected item correlation	Mean subscale score[b] (SD)	Internal consistency (Cronbach α)	Test-Retest (ICC)	≠	#	CFI	RMSEA	TLI
**Personalised Stigma**			4.86(2.56)	0.84 (95% CI; 0.81-0.86)	0.83 (95% CI; 0.71-0.90)	**0.357****	**0.327****			
Some people stop touching me soon they know/realise I am infected with HIV/AIDS	1.66(1.01)	0.65								
People I care for stopped calling me after knowing I suffer from AIDs.	1.63(1.00)	0.87								
I have lost friends for telling/explaining that I have AIDs.	1.59(0.96)	0.88								
**Disclosure Concerns**			8.74(2.37)	0.53 (95% CI; 0.45-0.60)	0.62 (95% CI; 0.36-0.77)	0.070	0.070			
Telling someone that I have AIDs is dangerous*	2.24(1.24)	0.83								
I do all I can to keep my AIDs (HIV) status secret	2.90(1.22)	0.46								
I am very careful to that person I tell about my HIV status (I am cautious/ very careful to (?of) the people I tell my HIV status)	3.60(0.78)	0.21								
**Concerns about Public Attitudes**			8.52(3.17)	0.83 (95% CI; 0.80-0.86)	0.79 (95% CI; 0.65-0.88)	**0.187****	**0.165****			
People who are suffering from AIDs are treated as if they are not like the other people.	3.05(1.18)	0.68								
People believe that a person infected with HIV is dirty.	2.74(1.26)	0.84								
Many people are worried when they are near a person infected with HIV.	2.75(1.22)	0.84								
**Negative Self Image**			6.32(3.00)	0.74 (95% CI; 0.70-0.80)	0.76 (95% CI; 0.60-0.86)	**0.372****	**0.330****			
I feel guilty because I am infected with HIV	2.11(1.23)	0.60								
People's attitudes about HIV/AIDs makes me feel very bad.	2.23(1.25)	0.78								
I feel I am not as good as others because I'm infected with HIV.	2.01(1.23)	0.73								
**Overall**			28.44(7.68)	0.80(95% CI; 0.77-0.83)	0.92(95% CI; 0.87-0.95)	**0.368****	**0.328****	**0.966**	**0.044**	**0.955**

Pearson product-moment correlation coefficient; **p<0.001; # correlation between HIV stigma and PHQ-9; ≠ correlation between HIV stigma and GAD-7

[a]Possible score for each item 1-4; higher scores reflect a higher level of perceived HIV-related stigma

[b]Possible score 3-12 on each sub-scale; higher scores reflect a higher level of perceived HIV- related stigma. [SD] Standard deviation. CFI = Comparative Fit Index. RMSEA = Root Mean Square Error of Approximation and TLI=Tucker Lewis Index.

For peer review only

For peer review only - http://bmjopen.bmj.com/site/about/guidelines.xhtml

Table 3: Univariate linear Regression of correlates of perceived HIV-related stigma among adults living with HIV from rural Kilifi

Independent variables	N	Personalised Stigma		Disclosure concerns		Public attitudes		Negative self-image		Overall HIV Stigma Score	
		B (95% CI)	p-value	B (95% CI)	p-value	B (95% CI)	p-value	B (95% CI)	p-value	B (95% CI)	p-value
Sociodemographic characteristics											
**Age**	450	-0.01 (-0.03, 0.02)	0.595	0.01 (-0.02, 0.02)	0.999	0.01 (-0.02, 0.04)	0.399	-0.01(-0.03, 0.03)	0.880	0.01 (-0.07, 0.08)	0.910
**Gender**	450		**0.080**		0.297		**0.004**		0.255		**0.011**
Male		Ref		Ref		Ref		Ref		Ref	
Female		**0.52(-0.06, 1.10)**		0.29 (-0.25, 0.83)		**1.07(0.35, 1.79)**		0.40 (-0.29, 1.08)		**2.27(0.54, 4.01)**	
**Marital Status**	450		**0.074**		**0.018**		0.350		**0.054**		**0.018**
Married		Ref		Ref		Ref		Ref		Ref	
Separated/Divorced/Widowed		**0.54(0.03, 1.04)**		**0.67(0.20, 1.14)**		0.25(-0.38, 0.87)		**0.73(0.14, 1.32)**		**2.18(0.67, 3.69)**	
Single/never married		**0.61(-0.14, 1.37)**		0.17 (-0.52, 0.87)		-0.43(-1.37, -0.51)		0.40 (-0.49, 1.28)		0.75(-1.50, 3.01)	
**Education Level**	450		0.424		**0.003**		**0.026**		**<0.001**		**<0.001**
Tertiary		Ref		Ref		Ref		Ref		Ref	
Secondary		-0.12(-1.36, 1.12)		0.08 (-1.06, 1.21)		0.68(-0.84, 2.21)		-0.03 (-1.46, 1.40)		0.61(-3.05, 4.26)	
Primary		-0.31(-1.43, 0.81)		0.48 (-0.55, 1.51)		**1.32(-0.06, 2.70)**		0.72 (-0.57, 2.01)		**2.20(-1.10, 5.51)**	
None		0.15(-1.01, 1.32)		**1.23(0.16, 2.30)**		**1.80(0.36, 3.23)**		**1.63(0.29, 2.97)**		**4.81(1.38, 8.25)**	
**Employment Status**	450		**0.191**		0.801		0.400		**0.071**		**0.098**
Formally Employed		Ref		Ref		Ref		Ref		Ref	
Self-employed		**0.67(-0.16, 1.50)**		0.27 (-0.50, 1.05)		0.46(-0.57, 1.49)		**0.73(-0.24, 1.70)**		**2.13(-0.36, 4.62)**	
Other		-0.67(-2.33, 0.99)		-0.02 (-1.57, 1.53)		-1.14(-3.20, 0.93)		-0.35 (-2.29, 1.60)		-2.17(-7.15, 2.81)	
Unemployed		**0.51(-0.25, 1.26)**		0.33 (-0.37, 1.03)		0.18(-0.76, 1.11)		**1.03(0.15, 1.91)**		**2.04(-0.22, 4.30)**	
**Currently living with**	450		0.575		0.714		0.974		0.889		0.897
Immediate family		Ref		Ref		Ref		Ref		Ref	
Relative/friend		0.86(-0.75, 2.47)		0.02(-1.47, 1.52)		0.18(-1.82, 2.18)		-0.45(-2.34,1.45)		0.62(-4.23, 5.46)	
Alone		0.01(-0.65, 0.66)		-0.25(-0.87, 0.36)		-0.06(-0.88, 0.76)		-0.07(-0.84, 0.70)		-0.38(-2.36, 1.60)	
**Asset index score [a] – mean (SD)**	450	**-0.12(-0.29-0.05)**	**0.171**	**-0.13(-0.29-0.03)**	**0.109**	-0.11(-0.33-0.10)	0.310	-0.12(-0.32-0.08)	0.244	**-0.48(-1.00-0.04)**	**0.068**
Clinical characteristics											
**BMI – kg/m^2, mean (SD), OM = 4**		0.004(-0.04, 0.05)	0.855	-0.03(-0.07,0.02)	0.244	0.03(-0.03,0.09)	0.309	-0.03(-0.18,0.12)	0.708	-0.03(-0.18,0.12)	0.708
**Viral Load OM = 145**	305		**0.183**		0.805		0.894		**0.033**		**0.173**
≤ 1000 copies/ml		Ref		Ref		Ref		Ref		Ref	
> 1000 copies/ml		**0.58(-0.28, 1.44)**		0.10(-0.70, 0.90)		0.07(-1.00, 1.14)		**1.05(0.08,2.02)**		**1.81(-0.79, 4.40)**	
Months since HIV diagnosis	450	0.00(-0.00, 0.01)	0.346	**-0.01(-0.01, -0.00)**	**<0.001**	-0.00(-0.01,0.00)	0.630	**-0.01(-0.01,0.00)**	**0.058**	-0.01(-0.03,0.00)	**0.091**
Months since cART initiation OM = 4	446	0.00(-0.00,0.01)	0.497	**-0.01(-0.01, -0.00)**	**0.001**	**-0.00(-0.01,0.00)**	**0.202**	**-0.01(-0.01, -0.00)**	**0.0308**	-0.02(-0.03, -0.00)	**0.031**
**Treatment characteristics**	450										
HIV status disclosure			0.651		**<0.001**		0.287		0.228		**0.023**

For peer review only

Yes		Ref		Ref		Ref		Ref		Ref	
No		0.23(-0.77,1.23)		**1.86(0.94,2.77)**		0.67(-0.57,1.91)		0.72(-0.45,1.89)		**3.47(0.49,6.46)**	
**Any current opportunistic infections**	450		**0.037**		0.768		0.759		**0.017**		**0.065**
No											
Yes		**0.65(0.04,1.26)**		0.09(-0.48,0.65)		0.12(-0.64,0.88)		0.87(0.16,1.59)		1.72(-0.11,3.55)	
**CMD comorbidity** OM = 48	402		**<0.001**		0.741		**0.145**		**<0.001**		**<0.001**
Absence		Ref		Ref		Ref		Ref		Ref	
Presence		**2.71(1.58, 3.84)**		0.18 (-0.91, 1.28)		**1.09(-0.38, 2.55)**		**3.07(1.76, 4.39)**		**7.06(3.71, 10.41)**	

Notes: Overall stigma scale represents the sum of all twelve items from the four subscales; A negative stigma score indicates less stigma. Bolded are variables taken into multivariate linear regression. *CMD* – symptoms of depression and anxiety, *BMI* body mass index, *WHO* world health organisation, *Ref* - Reference category, *OM* observation with missing value, cART combination antiretroviral therapy, *a* score range = 0 to 7, *b* score range = 12 to 48.

For peer review only

Table 4: Multivariate linear Regression of correlates of perceived HIV-related stigma among adults living with HIV from rural Kilifi

Independent variables	Dependent variables									
	Personalised stigma (n=402)		Disclosure concerns (n=450)		Public attitudes (n=450)		Negative self-image (n=402)		Overall HIV Stigma Score (n=402)	
	B (95% CI)	p-value	B (95% CI)	p-value	B (95% CI)	p-value	B (95% CI)	p-value	B (95% CI)	p-value
**Sociodemographic characteristics**										
**Gender**										
Male	Ref				Ref				Ref	
Female	**0.75(0.17, 1.34)**	**0.012**			**1.07(0.35,1.79)**	**0.003**			**2.00(0.21,3.80)**	**0.029**
**Education Level**										
Tertiary			Ref				Ref		Ref	
Secondary			-0.04(-1.14,1.07)	0.950			-0.05(-1.44,1.33)		-0.34(-3.83,3.16)	0.850
Primary			0.48(-0.52,1.48)	0.346			0.51(-0.73,1.74)		1.37(-1.75,4.50)	0.388
None			**1.24(0.20,2.28)**	**0.019**			**1.33**(0.04, 2.62)	**0.044**	**3.32(-0.01,6.65)**	**0.051**
**Clinical characteristics**										
Months since HIV diagnosis			**-0.01(-0.01, -0.00)**	**0.007**						
**Treatment characteristics**										
**HIV status disclosure**										
Yes			Ref						Ref	
No			**1.79(0.88,2.70)**	**<0.001**					**4.24(1.27,7.20)**	**0.005**
**CMD comorbidity**										
Absence	Ref						Ref		Ref	
Presence	**2.67(1.55, 3.79)**	**<0.001**					**3.04(1.74, 4.34)**	**<0.001**	**6.67(3.40,9.94)**	**<0.001**
Variance explained by the model Pseudo R-squared	**6.76%**		**8.66%**		**1.89%**		**7.71%**		**10.17%**	

Notes: Overall stigma scale represents the sum of all twelve items from the four sub-scales. Bolded are statistically significant values – symptoms of depression and anxiety, 95% CI - 95% confidence interval, Ref reference category

For peer review only

For peer review only - http://bmjopen.bmj.com/site/about/guidelines.xhtml

Table 5: Multi-Group Confirmatory Factor Analysis for age and gender sub-groups

Invariance Steps	Gender	RMSEA	TLI	CFI	ΔCFI	Age	RMSEA	TLI	CFI	ΔCFI
Configural Invariance	Female	0.051	0.934	0.950		Older adults	0.040	0.960	0.970	
	Male	0.051	0.934	0.950		Young Adults	0.040	0.960	0.970	
Metric Invariance	Female	0.052	0.932	0.943	***0.007***	Older adults	0.042	0.957	0.964	***0.006***
	Male	0.052	0.932	0.943	***0.007***	Young Adults	0.042	0.957	0.964	***0.006***
Scalar Invariance	Female	0.050	0.936	0.943	***0.000***	Older adults	0.041	0.959	0.963	***0.001***
	Male	0.050	0.936	0.943	***0.000***	Young Adults	0.041	0.959	0.963	***0.001***
Strict Invariance	Female	0.048	0.941	0.942	***0.001***	Older adults	0.041	0.959	0.960	***0.003***
	Male	0.048	0.941	0.942	***0.001***	Young Adults	0.041	0.959	0.960	***0.003***

Notes: CFI = Comparative Fit Index. RMSEA = Root Mean Square Error of Approximation and TLI=Tucker Lewis Index. Configural invariance - no constraints; Full metric invariance with all factor loadings constrained equal. Scalar invariance - with all intercepts constrained equal: Measurement invariance is assumed when ΔCFI is ≤0.01

For peer review only

For peer review only - http://bmjopen.bmj.com/site/about/guidelines.xhtml

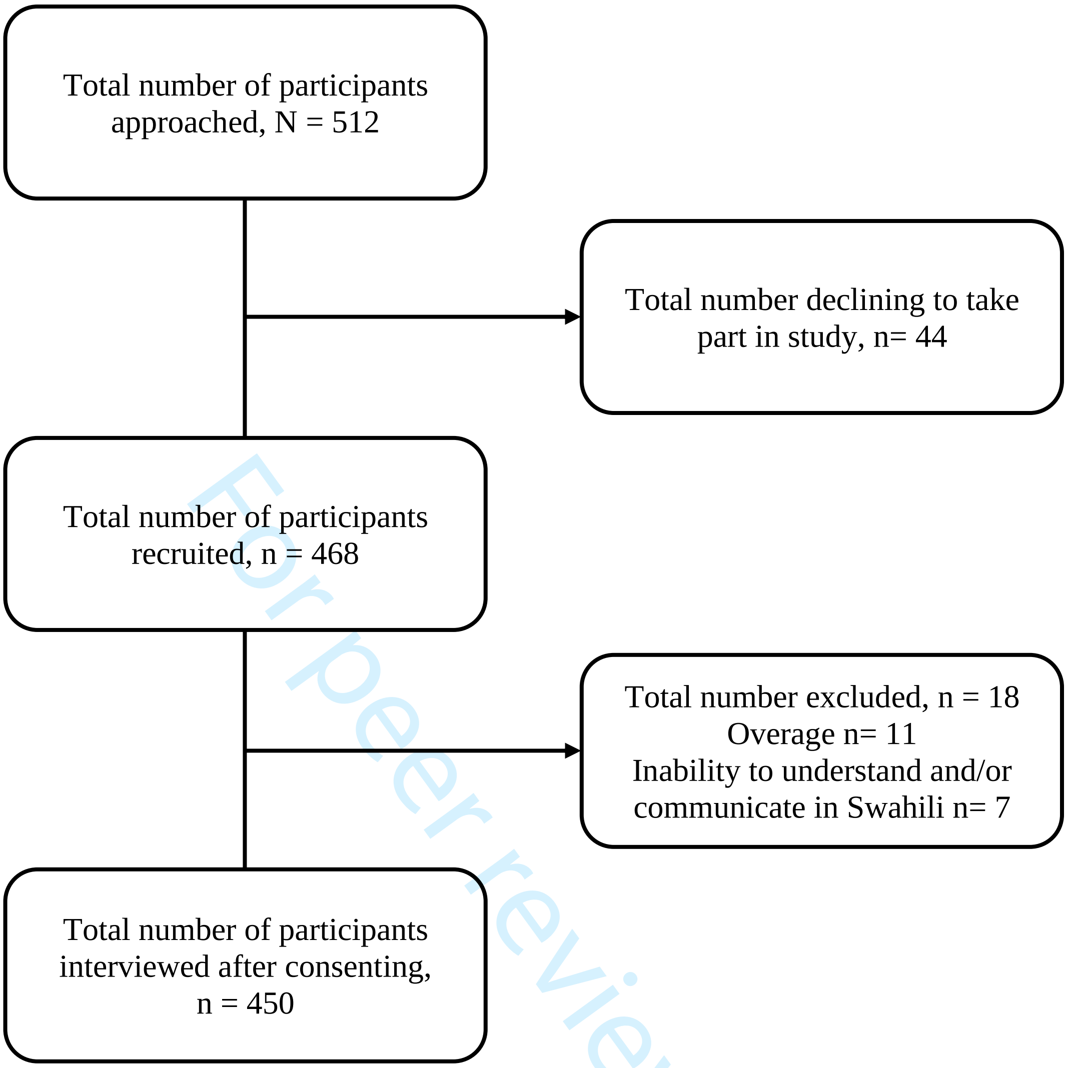

Figure 1: Study recruitment flow chart

For peer review only

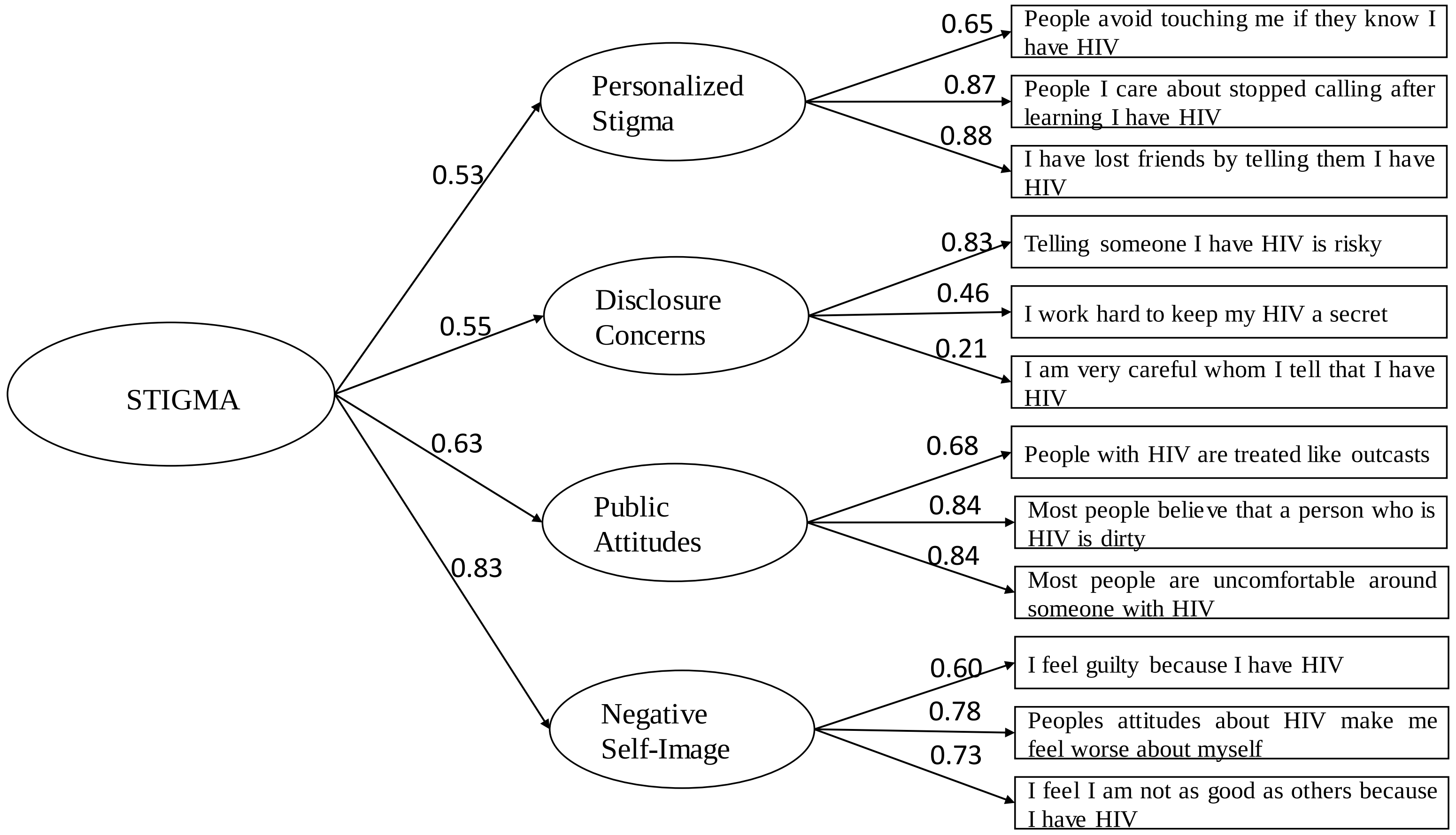

Supplementary Figure 1: Confirmatory factor analysis of the short version of the HIV Stigma Scale. Results show correlations between subscales (circles) and maximum likelihood estimates for the relation between subscales and items (rectangles). Sample (n = 435). Maximum likelihood estimates are standardised

STROBE 2007 (v4) Statement—Checklist of items that should be included in reports of *cross-sectional studies*

Section/Topic	Item #	Recommendation	Reported on page #
**Title and abstract**	1	(*a*) Indicate the study's design with a commonly used term in the title or the abstract	2
		(*b*) Provide in the abstract an informative and balanced summary of what was done and what was found	2
**Introduction**			
Background/rationale	2	Explain the scientific background and rationale for the investigation being reported	4-5
Objectives	3	State specific objectives, including any prespecified hypotheses	5
**Methods**			
Study design	4	Present key elements of study design early in the paper	5
Setting	5	Describe the setting, locations, and relevant dates, including periods of recruitment, exposure, follow-up, and data collection	5-8
Participants	6	(*a*) Give the eligibility criteria, and the sources and methods of selection of participants	6
Variables	7	Clearly define all outcomes, exposures, predictors, potential confounders, and effect modifiers. Give diagnostic criteria, if applicable	6-8
Data sources/ measurement	8*	For each variable of interest, give sources of data and details of methods of assessment (measurement). Describe comparability of assessment methods if there is more than one group	6-8
Bias	9	Describe any efforts to address potential sources of bias	6&8
Study size	10	Explain how the study size was arrived at	6
Quantitative variables	11	Explain how quantitative variables were handled in the analyses. If applicable, describe which groupings were chosen and why	9
Statistical methods	12	(*a*) Describe all statistical methods, including those used to control for confounding	8-10
		(*b*) Describe any methods used to examine subgroups and interactions	N/A
		(*c*) Explain how missing data were addressed	N/A
		(*d*) If applicable, describe analytical methods taking account of sampling strategy	8-9
		(*e*) Describe any sensitivity analyses	N/A
**Results**			

For peer review only

Participants	13*	(a) Report numbers of individuals at each stage of study—eg numbers potentially eligible, examined for eligibility, confirmed eligible, included in the study, completing follow-up, and analysed	29
		(b) Give reasons for non-participation at each stage - see details in figure 1	29
		(c) Consider use of a flow diagram	29
Descriptive data	14*	(a) Give characteristics of study participants (eg demographic, clinical, social) and information on exposures and potential confounders	10
		(b) Indicate number of participants with missing data for each variable of interest	23
Outcome data	15*	Report numbers of outcome events or summary measures	
Main results	16	(*a*) Give unadjusted estimates and, if applicable, confounder-adjusted estimates and their precision (eg, 95% confidence interval). Make clear which confounders were adjusted for and why they were included	25-27
		(*b*) Report category boundaries when continuous variables were categorized - see details in Table 1	23
		(*c*) If relevant, consider translating estimates of relative risk into absolute risk for a meaningful time period	N/A
Other analyses	17	Report other analyses done—eg analyses of subgroups and interactions, and sensitivity analyses	N/A
**Discussion**			
Key results	18	Summarise key results with reference to study objectives	10
Limitations	19	Discuss limitations of the study, taking into account sources of potential bias or imprecision. Discuss both direction and magnitude of any potential bias	15
Interpretation	20	Give a cautious overall interpretation of results considering objectives, limitations, multiplicity of analyses, results from similar studies, and other relevant evidence	12-15
Generalisability	21	Discuss the generalisability (external validity) of the study results	15
**Other information**			
Funding	22	Give the source of funding and the role of the funders for the present study and, if applicable, for the original study on which the present article is based	16

*Give information separately for cases and controls in case-control studies and, if applicable, for exposed and unexposed groups in cohort and cross-sectional studies.

Note: An Explanation and Elaboration article discusses each checklist item and gives methodological background and published examples of transparent reporting. The STROBE checklist is best used in conjunction with this article (freely available on the Web sites of PLoS Medicine at http://www.plosmedicine.org/, Annals of Internal Medicine at http://www.annals.org/, and Epidemiology at http://www.epidem.com/). Information on the STROBE Initiative is available at www.strobe-statement.org.

For peer review only

Measurement characteristics and correlates of HIV-related stigma among adults living with HIV: A cross-sectional study from coastal Kenya

Journal:	*BMJ Open*
Manuscript ID	bmjopen-2021-050709.R1
Article Type:	Original research
Date Submitted by the Author:	14-Sep-2021

Complete List of Authors:	Wanjala, Stanley; Ghent University Faculty of Medicine and Health Sciences, Public Health and Primary Care; Pwani University School of Humanities and Social Sciences, Social Sciences<br>Nyongesa, Moses; Centre for Geographic Medicine Research Coast, Clinical Neurosciences; Vrije Universiteit Amsterdam, Clinical, Neuro- and Developmental Psychology, Amsterdam Public Health Research Institute<br>Mwangi, Paul; Centre for Geographic Medicine Research Coast, Clinical Neurosciences<br>Mutua, Agnes M.; Centre for Geographic Medicine Research Coast, Clinical Neurosciences<br>Luchters, Stanley; Ghent University Faculty of Medicine and Health Sciences, Public Health and Primary Care; Aga Khan University - Kenya, Population Health<br>Newton, Charles; Centre for Geographic Medicine Research Coast, Clinical Neurosciences; Oxford University, Psychiatry<br>Abubakar, Amina A.; Centre for Geographic Medicine Research Coast, Clinical Neurosciences; The Aga Khan University - Kenya, Institute for Human Development

**Primary Subject Heading**:	HIV/AIDS
Secondary Subject Heading:	HIV/AIDS, Infectious diseases, Public health
Keywords:	HIV & AIDS < INFECTIOUS DISEASES, Epidemiology < INFECTIOUS DISEASES, PUBLIC HEALTH

I, the Submitting Author has the right to grant and does grant on behalf of all authors of the Work (as defined in the below author licence), an exclusive licence and/or a non-exclusive licence for contributions from authors who are: i) UK Crown employees; ii) where BMJ has agreed a CC-BY licence shall apply, and/or iii) in accordance with the terms applicable for US Federal Government officers or employees acting as part of their official duties; on a worldwide, perpetual, irrevocable, royalty-free basis to BMJ Publishing Group Ltd ("BMJ") its licensees and where the relevant Journal is co-owned by BMJ to the co-owners of the Journal, to publish the Work in this journal and any other BMJ products and to exploit all rights, as set out in our licence.

The Submitting Author accepts and understands that any supply made under these terms is made by BMJ to the Submitting Author unless you are acting as an employee on behalf of your employer or a postgraduate student of an affiliated institution which is paying any applicable article publishing charge ("APC") for Open Access articles. Where the Submitting Author wishes to make the Work available on an Open Access basis (and intends to pay the relevant APC), the terms of reuse of such Open Access shall be governed by a Creative Commons licence – details of these licences and which Creative Commons licence will apply to this Work are set out in our licence referred to above.

Other than as permitted in any relevant BMJ Author's Self Archiving Policies, I confirm this Work has not been accepted for publication elsewhere, is not being considered for publication elsewhere and does not duplicate material already published. I confirm all authors consent to publication of this Work and authorise the granting of this licence.

For peer review only

Measurement characteristics and correlates of HIV-related stigma among adults living with HIV: A cross-sectional study from coastal Kenya

Stanley W. Wanjala[1,2*], Moses K. Nyongesa[3,4], Paul Mwangi[3], Agnes M. Mutua[3], Stanley Luchters[1,5,6], Charles R.J.C. Newton[3,7,8], Amina A Abubakar[3,7,8,9]

[1]Department of Public Health and Primary Care, Ghent University, Ghent, Belgium

[2]Department of Social Sciences, Pwani University, Kilifi, Kenya

[3]Neuroassessment Group, KEMRI/Wellcome Trust Research Programme, Centre for Geographic Medicine Research (Coast), Kilifi, Kenya.

[4]Department of Clinical, Neuro- & Developmental Psychology, Vrije Universiteit Amsterdam, Amsterdam, Netherlands

[5]Department of Epidemiology and Preventive Medicine, Monash University, Melbourne, Australia

[6]Department of Population Health, Aga Khan University, Nairobi, Kenya

[7]Department of Public Health, Pwani University, Kilifi, Kenya

[8]Department of Psychiatry, University of Oxford, Oxford, UK

[9]Institute for Human Development, Aga Khan University, Nairobi, Kenya

Address of the corresponding author: Stanley W. Wanjala, Department of Social Sciences Pwani University, Box 195-80108, Kilifi, Kenya. Email: s.wanjala@pu.ac.ke

For peer review only - http://bmjopen.bmj.com/site/about/guidelines.xhtml

Abstract

Objective We studied the psychometric properties of the 12-item short version of the Berger HIV stigma scale and assessed the correlates of HIV-related stigma among adults living with HIV on the Kenyan coast.

Design Cross-sectional study.

Setting Comprehensive Care and Research Centre in the Kilifi County Hospital.

Participants Adults living with HIV on combination antiretroviral therapy were recruited and interviewed between February and April 2018 (n=450).

Main outcome measures HIV-related stigma

Results 450 participants with a median age of 43 years (interquartile range [IQR] = 36-50) took part in the study. Of these, 356 (79.1%) were female. Scale reliability and validity were high (alpha=0.80, test-retest reliability intraclass correlation coefficient =0.92). Using confirmatory factor analysis, we observed that the 12-item short version of the HIV stigma scale had a good fit for its hypothesised model (Comparative Fit Index =0.966, Tucker Lewis Index = 0.955, Root Mean Square Error of Approximation = 0.044). Multi-group confirmatory factor analysis indicated measurement invariance across gender and age groups as ΔCFI was ≤0.01. Multivariate linear regression established that being female *(β=2.001, 95%CI: 0.21, 3.80, p= 0.029),* HIV status non-disclosure *(β=4.237, 95%CI: 1.27, 7.20, p= 0.005)* and co-occurrence of depressive and anxiety symptoms *(β=6.670, 95%CI: 3.40, 9.94, p<0.001)* were significant predictors of perceived HIV-related stigma and that these variables accounted for 10.2% of the explained variability in HIV-related stigma among adults living with HIV from Kilifi.

Conclusions Our results indicate that the 12-item short version of the HIV stigma scale is a valid and reliable measure of HIV stigma in Kenya. Furthermore, our study indicates that interventions aimed at reducing stigma need to take into account gender to address the specific needs of women, people who have not disclosed their HIV status, and those exhibiting symptoms of depression and anxiety, thereby improving their quality of life.

Keywords: Adults**,** Stigma, Predictors, HIV/AIDS, antiretroviral therapy, Psychometrics, Kenya

For peer review only

Article Summary

Strengths and limitations of this study

- This is the first study to report the 12-item HIV stigma scale's measurement characteristics in the sub-Saharan African context.
- We report on the correlates of HIV stigma based on a culturally adapted measurement tool with good psychometric properties.
- We cannot generalise our findings to all adults living with HIV in Kenya as data were collected from one geographical setting and excluded adults older than 60 years.
- We cannot conclude how individuals experience stigma over time because of the study design limitation.

For peer review only

Introduction

HIV/AIDS remains a considerable public health concern globally, with sub-Saharan Africa (SSA) bearing the most HIV-related disease burden.[1] Despite SSA making up about 11% of the earth's population, it is the world's epicentre of HIV/AIDS. By the close of 2019, an estimated 38 million people were living with HIV globally, with an estimated 68% living in SSA, accounting for two-thirds of all individuals living with HIV.[1] Between 2010 and mid-2020, there has been an upsurge in the number of people accessing antiretroviral therapy (7.8-26 million).[1] Further, between 2010 and 2019, new HIV infections declined by an estimated 16% from 2.1 million/year to 1.7 million/year, and AIDS-related deaths dropped from 1.1 million to around 690,000 per year.[1] By the end of 2019, an estimated 1.5 million Kenyans were living with HIV, with 42,000 new infections and 21,000 AIDS-related deaths reported.[2] Estimates show that between 80% to 90% of the people living with HIV/AIDS (PLWHA) in Kenya are adults.[3] Additionally, 75% of adults in Kenya are reported to be on antiretroviral treatment.[2]

Erving Goffman[4] defined stigma as a process through which individuals are 'disqualified from full social acceptance' due to an undesirable 'mark' or 'label.' This label can either be a physical, health, or behavioural attribute that is regarded as 'deeply discrediting.'[4] In this study, the label is HIV seropositive status. Additionally, stigma, defined as a 'mark,' sets a person apart from others and links the person to undesirable characteristics such as stereotypes.[5] HIV-related stigma among PLWHA is prevalent throughout SSA.[6] HIV-related stigma has been identified as a severe obstacle in the way of effective responses to HIV.[7]

Although efforts have been scaled up to raise awareness and increase public knowledge about HIV since the epidemic started decades ago, social stigma is still associated with the disease.[8] Research has demonstrated that stigma keeps people from adopting HIV preventive behaviours and accessing needed care and treatment,[9] negatively impacting their health and well-being. Among women living with HIV, the decision to disclose their HIV seropositive status is likely affected by perceived stigma.[10]

From previous research, HIV stigma experienced by PLWHA can either be enacted, anticipated, or internalised.[11] Enacted stigma includes an individual's experiences, prejudice, and/or discrimination from others because of one's HIV status. Anticipated stigma includes an individual's expectation of experiencing enacted stigma, while internalised stigma refers to the extent to which PLWHA have adopted negative feelings and beliefs about PLWHA.[12]

For peer review only - http://bmjopen.bmj.com/site/about/guidelines.xhtml

A variety of instruments designed to measure HIV-related stigma have been published.[13-21] Berger's 40-item HIV stigma scale (HSS-40) is the most commonly used instrument and one of the few instruments covering all stigma mechanisms affecting PLWHA.[12] It takes up to 25 minutes to complete the HSS-40[22], which may limit its application, especially in extensive surveys. Though shortened versions covering 25[22] and 32[23] items of the HIV stigma scale exist, the 12-item HIV stigma scale (HSS-12)[14] version of the Berger HIV stigma scale was examined in the present study as it facilitates the inclusion of HIV stigma in more extensive surveys. Furthermore, it has comparable psychometric properties to the full-length scale.[14] While evidence from other parts of the world[14] indicates that the HSS-12 is psychometrically sound, we are unaware of any study that has reported this scales' psychometric properties in the SSA context.

Empirical evidence indicates that sociodemographic characteristics such as age,[24 25] gender,[25-27] employment,[28] educational attainment,[29-31] and marital status,[32] are significantly correlated with HIV-related stigma. However, the directionality is inconsistent. An explanation for the different findings regarding correlates and predictors of HIV-related stigma might be due to the diverse research strategies applied and the sample composition. Research shows that stigma and disclosure of HIV status are interrelated phenomena for people living with HIV/AIDS.[33] Furthermore, persons who have not disclosed their HIV status exhibit higher levels of perceived HIV-related stigma and greater levels of concern about HIV disclosure.[34]

Despite the abundance of published reports on HIV-related stigma and its predictors amongst specific sub-groups of the adult population, there is a paucity of research findings focusing on predictors of HIV-related stigma across the entire adult population. Further, no study in the SSA context has tested for the validity and reliability of the HSS-12. This study aims to determine the correlates of HIV-related stigma among adults living with HIV from Kilifi, Coastal Kenya. Specifically, the study aims to: i) examine the psychometric properties of the 12-item Berger Stigma Scale; and ii) establish the correlates of stigma among adults living with HIV in Kilifi.

Methods

Study setting

This cross-sectional study was conducted at the Kenya Medical Research Institute-Wellcome Trust Research Programme (KEMRI-WTRP), Centre for Geographic Medicine

For peer review only - http://bmjopen.bmj.com/site/about/guidelines.xhtml

Research(Coast), Kilifi, Kenya. It was based at the Comprehensive Care and Research Centre (CCRC) in the Kilifi County Hospital (KCH). The majority of Kilifi County residents are poor (71.4% live below the poverty line), lack formal education, and earn a living mainly through subsistence farming or fishing.[35-37] HIV prevalence in adults is estimated to be at 4.5%.[38] The CCRC offers clinical services such as management of opportunistic infections, HIV testing and counselling, family planning, nutritional counselling, cervical cancer screening, the dispensation of antiretroviral therapy (ART), and serves as a research facility. About 60 patients are seen daily. By 2020, the clinic has enrolled over 9,000 patients of all ages.

Study participants

This data is part of a larger project focusing on diverse outcomes in adults living with HIV, including mental health and health-related quality of life. A cross-sectional survey of 450 study participants among patients attending an HIV care and treatment clinic at Kilifi County Hospital was conducted between February and April 2018 (Figure 1). The participation criteria were age (18-60 years old) with confirmed HIV positive status, on combination antiretroviral therapy, and informed consent to participate. Participants with an acute medical illness or cognitive difficulties at the time of enrolment/administration of questionnaire or could not understand and/or communicate in the national language (Kiswahili), which was used during the administration of all study instruments, were excluded. A research team member introduced the study to eligible participants when they visited the clinic for scheduled appointments. Those who consented to take part responded to the instruments at the clinic.

Data Collection Procedures

Study data were collected and managed using REDCap electronic data capture tools hosted at KEMRI Wellcome Trust Programme[39 40]. REDCap (Research Electronic Data Capture) is a secure, web-based software platform designed to support data capture for research studies, providing 1) an intuitive interface for validated data capture; 2) audit trails for tracking data manipulation and export procedures; 3) automated export procedures for seamless data downloads to common statistical packages; and 4) procedures for data integration and interoperability with external sources. Data collection instruments were interviewer-administered via android tablets, in the same order, and under the same administration environment. Research assistants underwent a 4-day training in research ethics and proper interviewing techniques (with role-plays) and were familiarised with the tablet-based

For peer review only - http://bmjopen.bmj.com/site/about/guidelines.xhtml

questionnaires. The questionnaire administration took place in a quiet and private room within the CCRC in KCH, and the interview session lasted between 30 to 45 minutes.

Measures

HIV-related stigma: The short version (HSS-12) of the Berger HIV stigma scale[14] was used to assess patient-perceived HIV-related stigma under four dimensions: i) *personalised stigma*; ii) *disclosure concerns*; iii) *negative self-image;* and iv) *concerns with public attitudes,* each comprising a sub-scale of the instrument. *Personalised stigma* has been suggested to represent the enacted stigma mechanism, *disclosure concerns,* and *concerns with public attitudes* dimensions have been proposed to represent anticipated stigma mechanism, and *negative self-image* has been proposed to represent internalised stigma mechanism.[12] Items on this scale are rated from 1-4, with (1) being "strongly disagree" and (4) "strongly agree." The possible score for each item ranges from 1 to 4 (3–12 for sub-scale), and a total score ranges between 12 and 48 and is derived from the summation of item scores. Higher scores designate a greater level of perceived HIV-related stigma.

Patient Health Questionnaire version 9 (PHQ-9)[41] was administered as a measure of depressive symptoms. The PHQ-9 is a nine-item scale rated on a Likert-type scale ranging from 0 "not at all" to 3 "nearly every day." Item scores are summated to derive a total score ranging from 0 to 27. It has previously been found to have good internal consistency (Cronbach alpha 0.78) and acceptable test-retest reliability (intraclass correlation coefficient [ICC]=0.59) when used among adults living with HIV infection in Kenya.[42]

Generalised Anxiety Disorder (GAD-7)[43] was administered as a clinical measure for assessing generalised anxiety disorder based on DSM-IV criteria. The GAD-7 is a seven-item self-report instrument rated on a Likert-type scale ranging from 0 "not at all" to 3 "nearly every day." The scale score ranges from 0 to 21. There is evidence in support of the reliability and validity of this scale in Kenya.[44] Scores from PHQ-9 and GAD-7 were combined to generate a variable called CMD (symptoms of common mental disorders) comorbidity, indicating the co-occurrence of depressive and anxiety symptoms.

Sociodemographic and asset index items: A sociodemographic questionnaire was used to collect information on the participants' age, gender, relationship status, educational level, employment status, and whom they currently shared a residence. Furthermore, an asset index previously used in this setting[45] was used to collect information about participants' socio-

For peer review only - http://bmjopen.bmj.com/site/about/guidelines.xhtml

economic status (SES) based on disposable assets owned. Participants were asked for ownership of disposable items such as radio, television, refrigerator, gas, bicycle, motorcycle, and car. The final SES score had seven (7) items. A total asset score is calculated, and higher scores indicate a better SES. The maximum possible score for the asset index score was 7. An asset index to estimate family wealth has been recommended as an alternative approach to estimating SES in settings where reliable data on family income may not be available.[46]

Clinical information: Participants' data were extracted from the clinic's medical record database and filled into a clinical record form. This information included participants' dates of HIV-diagnosis, combination antiretroviral therapy initiation, most current combination antiretroviral therapy regimen, cluster of differentiation 4 (CD4) cell count, viral load (within the last one year), recent height and weight (for Body Mass Index (BMI) calculation), and data on World Health Organization (WHO) clinical staging. Participants' clinical information was retrieved from their clinical records after consent was granted. Patient-unique clinic numbers were used to access participants' medical records. We report substantial missing participant data on viral load from the database (n = 145) with no follow-up record of CD4 cell count for all study participants.

Instrument translation and cross-cultural adaptation

The English version of the HSS-12 was forward translated by two independent bilingual translators to Kiswahili and back-translated into English by two independent back translators (oblivious of the original version). A group of Kenyan HIV researchers bilingual and fluent in both Kiswahili and English and the translators had a harmonisation meeting to review the content, conceptual, semantic, and idiomatic equivalence of the questionnaires to ensure the cultural relevance of the HSS-12. Before conducting the formal phase of the study, fifteen pretest interviews were conducted to assess instrumentation rigour and develop measures to address any limitations or threats to bias and management procedures. The final version of the questionnaire was obtained after the incorporation of changes emerging from pretesting. Pretesting procedures have been elaborated further elsewhere.[47]

Patient and public involvement

Patients were not involved in the design and conduct of this study.

For peer review only - http://bmjopen.bmj.com/site/about/guidelines.xhtml

Statistical analyses

Factor structure and measurement invariance across age-groups and gender

First, Confirmatory Factor Analysis (CFA) was used to examine the HIV-stigma scale's factor structure. A CFA model representing the Swahili version of the HSS-12 was set up and analysed with weighted least square mean and variance adjusted (WLSMV) using the lavaan[48] package in R statistical software[49] on all the 450 observations. The Goodness of fit was assessed using the χ^2 test, Comparative Fit Index (CFI), Tucker Lewis Index (TLI), and root mean square error of approximation (RMSEA). The data was expected to have a good fit to the model if the χ^2 test was non-significant, CFI and TLI values were greater than 0.90, and RMSEA score was lower than 0.05.[50]

Secondly, after defining the model, Multi-Group Confirmatory Factor Analysis (MGCFA)[51] was used to test for measurement invariance of the HSS-12 for gender and age groups. Change in CFI (ΔCFI) has been suggested as a robust statistic for testing the between-group invariance of CFA models. Additionally, it has been recommended that invariance can be assumed when ΔCFI is ≤0.01 in absolute values.[52]

Internal construct validity and convergent validity

Means and standard deviations were used to evaluate the distribution of scores within the subscale and among the items. Itemised means and standard deviations were expected to be almost the same within the subscale, justifying item scores' aggregation into subscale scores.[53] The item-total correlation was used to evaluate internal construct validity. Each items' corrected item-total correlation coefficients were calculated and expected to exceed 0.4 and vary in range. Convergent validity was assessed using the Pearson correlation coefficient between HSS-12, PHQ-9, and GAD-7 scores. Correlation coefficients were interpreted as small (0.10–0.29), moderate (0.30–0.49), and large (0.49 and above).[54]

Reliability

Cronbach's alpha and ordinal alpha were used to examine each subscale's internal consistency and overall scores of the Swahili version of the HSS-12. Cronbach's alpha was considered acceptable if greater than (>0.7).[55] The intra-class correlation coefficient (ICC) was used to examine test-retest of the Swahili version of the HSS-12 by correlating scores taken at two different time points (2 weeks apart)[56] using the same measure administered to the same participant. ICC of 0.60 was considered marginal, 0.70 acceptable, and anything over 0.80 considered high.[57]

For peer review only - http://bmjopen.bmj.com/site/about/guidelines.xhtml

Sample characteristics and correlates

Frequencies and means (with percentages and standard deviations) were used to describe sample characteristics. Univariate and multivariable linear regression were used to assess factors associated with both stigma subscales and the overall stigma scale. In the regression model, stigma scores were expressed as a continuous measure. Independent variables included age, gender, marital status, education level, employment status, socioeconomic status (SES), body mass index (BMI), viral load, WHO clinical stages, months since HIV diagnosis, months since cART initiation, HIV status disclosure, self-reported opportunistic infections, and the co-occurrence of depressive and anxiety symptoms. Our review of the literature informed factors included in the model. All variables with $p<0.20$ were included in the multivariable regression model apart from viral load because participants had missing values (n=145). The final multivariable models were generated using a backward stepwise approach by eliminating all variables independently with $p>0.05$. Assumptions of linear regression testing were visually inspected through histograms (linearity), normal probability plots (normality), and plots of residual versus predicted values (homoscedasticity). Multicollinearity was assessed using the variance inflation factor (VIF). There were no multicollinearity problems. Modelling was undertaken five times in total: once to predict overall stigma and once to predict each of the four subscales. R (version 4.0.2) statistical software package[49] was used to explore the construct validity of the HSS-12. All other analyses were run using (Stata version 14.0) statistical software package.[58]

Results

Sample Characteristics

The 450 participants had a median age of 43 years (IQR = 36-50), ranging from 18 to 60 years. The vast majority of the sample were female (79.1%), had attained basic primary level education (53.1%), lived with a family member (82.4%), and were unemployed (59.8%). Less than half of the study participants (43.8%) were separated, divorced, or widowed. The mean BMI was within the normal range (mean [SD] = 22.4 [4.8]). Most study participants had disclosed their HIV status to others (94.0%). The median time since HIV diagnosis was 8.8 years (IQR = 4.67-11.50), ranging from 0 to 18 years. A total of 417(93.7%) were in stage 1 of the WHO clinical staging, and 425 (95.3%) were on the first-line cART regimen (Table 1). The median time elapsed since cART initiation was 6.7 years (IQR = 3.67-10.00). At the time of the interview, less than a fifth (18.4%) of the study participants had an opportunistic infection.

For peer review only - http://bmjopen.bmj.com/site/about/guidelines.xhtml

Perceived overall stigma scores ranged from 12 to 48, with a median score of 28 (IQR = 23-33). Using PHQ-9 and GAD-7 cut-off score of ≥10, which has been shown to maximise specificity and sensitivity for depression[59] and general anxiety disorder[43] screening, the overall prevalence of depression and anxiety was 13.8% and 5.3%, respectively, among enrolled participants. The co-occurrence of depressive and anxiety symptoms was present in 4.7% of the study participants.

Factor structure and measurement invariance across age groups and gender

Supplementary Supplementary Figure 1 presents CFA results with standardised correlation coefficients. Our hypothesised model that the overall stigma scale comprises four sub-scales correlated was confirmed given the observed fit indexes. The χ^2 test was statistically significant (χ^2 = 91.982, df= 50, p=0.000) but alternate fit measures indicated acceptable fit; RMSEA: 0.044; CFI:0.966 and TLI: 0.955. These results generally indicate that the data had a good fit to the model and that we can confidently use both total and sub-scale scores in this population. Measurement invariance across age groups and gender was supported because ΔCFIs are lower than 0.01 in all models suggesting that measurement invariance can be assumed.

Internal construct validity and convergent validity

The factor loading of all items on the hypothesised scale was good except for item 6 (0.21) under the disclosure concern subscale. Convergent validity of the HSS-12 was demonstrated by the small to moderate correlations between HSS-12 and the correlation with the following relevant measures: GAD-7 (r =0.368, p<0.001) and PHQ-9 (r =0.328, p<0.001) Table 2.

Reliability: Internal consistency and test-retest

Cronbach's alpha (α) for the subscales and overall scale were all >0.7 (see Table 2) except for the disclosure concern sub-scale, which was 0.53 (95%CI: 0.46, 0.60). Additionally, ordinal α for the subscales ranged from 0.65-0.91. The test-retest reliability of the short 12-item version of the HIV stigma scale was excellent, 0.92 (95%CI: 0.87, 0.95). Additionally, Table 2 presents descriptive statistics for the stigma scale on the item level and subscale level. Corrected item-total correlation coefficients were >0.4 for all the items apart from one item (0.21) in the disclosure concerns subscale. A variation of 0.46-0.88 indicates that the intended stigma concepts' broadness had been captured.

For peer review only - http://bmjopen.bmj.com/site/about/guidelines.xhtml

For peer review only

292 **Correlates of perceived HIV-related stigma**

293 Table 3 and Table 4 present results based on univariate and multivariable regression analyses, respectively. In the univariate model, it was found that being female, being separated, divorced or widowed, having primary or no level of education, being self-employed or unemployed, having a low asset index score, having a viral load of >1000 copies/ml, decreased duration since HIV diagnosis, decreased duration since cART initiation, HIV status non-disclosure, having any current opportunistic infection and co-occurrence of depression and anxiety symptoms were significantly associated with overall HIV stigma scores.

300 *Personalised stigma* was significantly associated with being female, being single, separated, divorced or widowed, self-employed or unemployed, having a low asset index score, having a viral load of >1000 copies/ml, having any current opportunistic infection, and the co-occurrence of depressive and anxiety symptoms. *Disclosure concern* was significantly associated with being separated, divorced or widowed, having no level of education, having a low asset index score, less time elapsed since HIV diagnosis, less time elapsed since cART initiation, and HIV status non-disclosure. *Concern with public attitudes* was significantly associated with being female, having primary or no level of education, decreased duration since cART initiation, and the co-occurrence of depressive and anxiety symptoms. *Negative self-image* was significantly associated with being separated, widowed or divorced, having no level of education, being self-employed or unemployed, having a viral load of >1000 copies/ml, decreased duration since HIV diagnosis, decreased duration since cART initiation, having any current opportunistic infection and the co-occurrence of depressive and anxiety symptoms.

313 When a multiple linear regression model was run, it was found that being female (β=2.001, *95%CI: 0.21, 3.80,* p=0.029), HIV status disclosure (β=4.237, 95%CI: 1.27, 7.20, p=0.005) and co-occurrence of depressive and anxiety symptoms (β=6.670, *95%CI: 3.40, 9.94,* p<0.001) were significant predictors of perceived HIV stigma. Having no education was associated with increasing stigma levels at p=0.051 (β=3.318, *95%CI: -.01, 6.65*). Regression results indicated that the model explained 10.2% of the variance and that the model was a significant predictor of perceived HIV stigma F (6, 395) = 7.46, p<.001).

320 Concerning the four subscales, we found that *personalised stigma* was positively correlated with being female and the co-occurrence of depressive and anxiety symptoms. *Disclosure concern* was inversely correlated with duration since HIV diagnosis and positively correlated with having no level of education and HIV status non-disclosure. *Concerns with public*

For peer review only - http://bmjopen.bmj.com/site/about/guidelines.xhtml

attitudes were positively correlated with being female. *Negative self-image* was positively correlated with having no level of education and the co-occurrence of depressive and anxiety symptoms.

Discussion

This cross-sectional analysis of data from adults living with HIV observed that the HSS-12 presents excellent psychometric properties. Additionally, we observed that stigma was associated with both physical and mental well-being. According to our study, correlates of HIV-related stigma include being female, HIV status non-disclosure, and the co-occurrence of depressive and anxiety symptoms.

Factor structure, measurement invariance, validity and reliability of the short 12-item Swahili version of the HIV Stigma Scale

The study examined the stigma scale's psychometric properties to assess its usefulness and describe the correlates of HIV-related stigma among adults living with HIV in Kilifi. Reliability and validity were acceptable, and confirmatory factor analysis supported the four-factor solution measuring the four dimensions of HIV stigma. Cronbach's alpha for the HSS-12 among the Kenyan population is similar to the Swedish population in which the scale was developed.[14] Although Cronbach's alpha for the adapted HSS-12 sub-scales was slightly lower (0.53-0.84) than the initial version of HSS-12 (0.80-0.88), its' alpha for the total scale was 0.80 suggesting good internal consistency. Furthermore, the adapted HSS-12 had an ordinal alpha of 0.86. The difference between ordinal alpha and Cronbach's alpha values could be attributed to high skewness and kurtosis values for some of the questionnaire's questions, influencing Cronbach's alpha estimate values.[60 61]

Measurement invariance of the Swahili HSS-12 was evaluated and confirmed across main interest groups: gender and age. Our results indicated that the measurement model of the Swahili HSS-12 as a patient-reported outcome to measure perceived HIV stigma among adults is comparable across age groups and gender (Table 5).

Test-retest reliability, an indicator of scale stability over time, was of acceptable levels. The original HSS-40 has been used in diverse settings[13 62] among adults 18 years and above, reporting a test re-test reliability between (ICC=0.89-0.92). To the best of our knowledge, no study has reported the test re-test reliability of the HSS-12.

For peer review only - http://bmjopen.bmj.com/site/about/guidelines.xhtml

We examined the construct validity of the scale using CFA since its hypothesised structure has been published.[14] Our results indicated that the hypothesised model fit the data well and was almost similar to what was reported by a study conducted in Sweden.[14] These results indicate that one can use both the total scores and the subscale scores and interpret the results in confidence, knowing that the items fit well together. HSS-12 evidenced convergent validity by being correlated with PHQ-9, a measure of depression, and GAD-7, a measure of anxiety in conventional ways.

The HSS-12 was reliable and valid for detecting stigma among adults living with HIV at the Kenyan Coast. Consequently, HSS-12 can be practically used as a brief screening tool for stigma-related problems both for research and clinical purposes. Future research could examine its predictive validity and evaluate its sensitivity to changes. This information would be crucial in determining its usefulness as an evaluation tool for programmes and interventions.

Correlates of Stigma

Being female was positively associated with increased perceived HIV-related stigma scores, *personalised stigma,* and *concern with public attitudes*. This finding agrees with previous studies from SSA[63] and outside[64 28] that reported a positive association between female gender and perceived HIV-related stigma. Research shows that females are more likely to suffer from stigma in patriarchal societies like Kenya than males.[65 66] Research has established that the African society is less tolerant of females living with HIV than males living with HIV.[67 68] Due to women's subordinate status in society, they are often stigmatised as vectors of transmission.[69] Furthermore, the common belief that HIV is caused by indecent sexual behaviour has worse societal consequences for women who are expected to be monogamous, unlike men in most African societies.[67] Women are often blamed counterfactually to be responsible for HIV transmission.[67] Similar processes can be assumed to be at work in the Kenyan coastal region.

HIV status disclosure was positively associated with overall HIV-related stigma scores and *disclosure concerns,* with persons who had not disclosed their HIV status reporting greater levels of concern about HIV disclosure concerns. Anakwa and colleagues found that PLWHA with higher levels of perceived HIV-related stigma reported greater levels of HIV disclosure concerns; therefore, they are less likely to disclose their status.[34] From our study, only 6% had not disclosed their status to anyone. HIV status non-disclosure might be a protective behaviour for PLWHA to conceal their status, evade adverse reactions towards themselves, weigh other

For peer review only - http://bmjopen.bmj.com/site/about/guidelines.xhtml

people's reactions, and as a sign of concern about the implication of their disclosure on their disclosure targets.[70 71] Furthermore, disclosure entails deciding how and to whom to disclose and identifying appropriate opportunities to disclose or devising means to conceal ones' status and/or medication in order to improve access and adherence to their treatment regimen.

The co-occurrence of depressive and anxiety symptoms was positively correlated with overall HIV-related stigma scores, *personalised stigma,* and *negative self-image.* This finding corroborates previous studies among PLWHA carried out within SSA,[30 72, 26] and outside,[73 74] which have invariably found a significant association between HIV-related stigma and depressive symptoms. Liu and colleagues[75] reported that the more stigma PLWHA perceived, the more anxiety they experienced. Similarly, we report that HIV-related stigma is significantly associated with the co-occurrence of depressive and anxiety symptoms. Additionally, an individual's perception of themselves in light of their diagnosis appears to trigger depression.[76] Screening for depression, anxiety, and HIV-related stigma might provide insights on interventions that may promote a positive attitude and self-image, thereby reducing depression, anxiety, and stigma, leading to psychological and physical well-being. Given the cross-sectional nature of the study, we cannot claim causality. However, the association between co-occurrence of depressive and anxiety symptoms and stigma provides the impetus for: a) longitudinal studies to elucidate causal pathways; and b) targeted interventions to address both stigma and mental health to improve health outcomes of adults living with HIV.

Other factors influencing the four subscales were also established. Having no level of education was positively associated with higher reported *disclosure concerns* and *negative self-image,* corroborating findings of studies carried out in Nigeria[77] and the USA.[78] Lower levels of education may lead to less exposure, lack of or little knowledge about HIV infection and transmission. In contrast, higher levels of education might lead to higher levels of knowledge, providing exposure to new ways of thinking and new sources of information about the HIV pandemic resulting in the reduction of less supportive attitudes towards PLWHA.[79 80] Previous research has demonstrated that people with high levels of knowledge of the transmission routes for HIV consistently had more supportive attitudes towards those with HIV demonstrating the role that knowledge has in reducing the misconceptions that act to create fear and shape stigma.[79]

Months since HIV diagnosis was inversely associated with *disclosure concerns,* with persons with a more recent diagnosis reporting greater levels of concern about HIV status disclosure.

For peer review only - http://bmjopen.bmj.com/site/about/guidelines.xhtml

This is consistent with a study of people living with HIV/AIDS (PLWHA) in China[81] and among African Americans.[78] This finding suggests that living longer with HIV is associated with positive outcomes because PLWHA are likely to adjust over time to their HIV positive status, receive more information, develop greater insights and understanding of the disease and establish psychological mechanisms to better cope with HIV stigma leading to lower levels of perceived HIV stigma.

Strengths and limitations of this study

A potential strength is that this is the first study to report the measurement characteristics of the 12-item HIV stigma scale in the SSA context. We recognise several potential limitations in this study. First, the study was in a clinical setting where our study sample consisted of adults living with HIV on cART. Compared to untreated individuals living with HIV, it is likely that levels of HIV stigma would be lower in our sample because it has been shown that access to ART lowers stigma.[82-84] Second, this study is cross-sectional, so causality for the observed significant associations cannot be inferred. We can also not conclude how individuals may experience stigma over time because of the study design limitation. Third, findings may not be generalisable to all adults living with HIV in Kenya as data were collected from one geographical setting and excluded adults older than 60 years. Fourth, because many participants (n = 145) lacked information on their most recent viral load and none had follow-up data on CD4 counts, these variables were excluded from the regression analyses. A disproportionately large number of patients, combined with financial constraints, may explain why these tests are not routinely performed. Future studies, particularly those from resource-constrained settings, should budget for these tests because these biological factors have been associated with HIV-related stigma.[85] Finally, the psychometric robustness of the disclosure concern sub-scale may be limited. We recommend further research into investigating this specific subscale.

Conclusions and implications

From the study, the 12-item short version of the Berger HIV stigma scale[14] had good psychometric properties and can be recommended for research purposes. The current study suggests that women, those who have not disclosed, and those experiencing co-occurring depressive and anxiety symptoms experience a higher level of perceived HIV stigma in Coastal Kenya. This finding is useful in designing future interventions to improve the quality of life of people living with HIV/AIDS. We propose interventions that need to take into account gender

For peer review only - http://bmjopen.bmj.com/site/about/guidelines.xhtml

to address the specific needs of women, people who have not disclosed their HIV status, and those exhibiting symptoms of depression and anxiety, thereby improving their quality of life. All these interventions will help in bettering both the physical and mental well-being of adults living with HIV. Additionally, it would be prudent to investigate further the association between lower education and HIV-related stigma as we found a marginal association.

Acknowledgements

We thank our participants for taking the time and effort to participate in this study and the Comprehensive Care and Research Centre (CCRC) staff in the Kilifi County Hospital HIV care and treatment clinic for their unrelenting support. We appreciate Patrick Katana Vidzo for helping with the entry of clinical data. The authors acknowledge the Kenya Medical Research Institute (KEMRI) Director for permission to publish this work.

Footnotes

Author Contributions: SWW, CN, and AA conceptualised the study. SWW, MKN and AA designed the study. PM formulated study questions for tablet administration and managed the data. SWW and MKN supervised data collection. SWW, MKN, and AM participated in data collection. SWW and MKN analysed the data. SWW, MKN, PM, AM, SL, CN and AA contributed to interpreting the data. SWW wrote the first draft of the manuscript. All authors reviewed subsequent versions of the manuscript and approved the final version for submission. The corresponding author affirms that all listed authors meet authorship criteria and that no other author meeting the criteria has been omitted.

Funding: This work was supported by funding from the Medical Research Council (Grant number MR/M025454/1) to AA. This award is jointly funded by the UK Medical Research Council (MRC) and the UK Department for International Development (DFID) under the MRC/DFID concordant agreement and is also part of the EDCTP2 program supported by the European Union.

Disclaimer: The funders had no role in the design, collection, analysis, interpretation and manuscript writing.

Competing interests: None declared.

For peer review only

Ethical approval: The local institutional review board, Scientific and Ethics Review Board (SERU; Ref KEMRI/SERU/CGMR-C/108/3594), granted ethical approval to recruit participants into the study. We obtained authorisation to work in the HIV care and treatment clinic from the Ministry of Health, County government of Kilifi (Ref HP/KCHS/VOL.VIX/65). Study participants provided written, informed consent to be part of the study.

Transparency: The lead author (SWW) confirms that the manuscript is an honest, accurate, and sincere account of the research being reported; no important aspects of the research have been omitted; and that explanations for any discrepancies from the research as planned (and, if relevant, registered) have been provided.

Data sharing statement: No additional data are available. Anyone interested in accessing the data reported in this article is free to write to the Data Governance Committee of the KEMRI Wellcome Trust Research Programme, review the application and advise as appropriate, and ensure that uses are compatible with the consent obtained from participants for data collection. Requests can be sent to the coordinator of the Data Governance Committee using the following email: dgc@kemri-wellcome.org.

For peer review only

REFERENCES

1. Joint United Nations Programme on HIV/AIDS. Global HIV & AIDS statistics — 2020 fact sheet [Available from: https://www.unaids.org/en/resources/fact-sheet accessed 22/2/2021.
2. Joint United Nations Programme on HIV/AIDS. UNAIDS DATA 2020. Switzerland: UNAIDS; 2020. Available from: https://www.unaids.org/sites/default/files/media_asset/2020_aids-data-book_en.pdf [Accessed 8/9/2021]
3. National AIDS Control Council (NACC). Kenya AIDS Response Progress Report 2014: Progress towards Zero. Nairobi, Kenya: National AIDS Control Council of Kenya; 2014. Available from: https://www.unaids.org/sites/default/files/country/documents/KEN_narrative_report_2014.pdf [Accessed 04/06/2020]
4. Goffman E. Stigma. Notes on the Management of Spoiled Identity. New York: Simon and Shuster: Inc, 1963.
5. Jones EE, Farina A, Hastorf AH, et al. Social stigma: the psychology of marked relationships. New York: W.H. Freeman 1984.
6. Maluccio JA, Wu F, Rokon RB, et al. Assessing the Impact of Food Assistance on Stigma Among People Living with HIV in Uganda Using the HIV/AIDS Stigma Instrument-PLWA (HASI-P). *Aids Behav* 2017;21(3):766-82. doi: 10.1007/s10461-016-1476-9 [published Online First: 2016/07/04]
7. Dahlui M, Azahar N, Bulgiba A, et al. HIV/AIDS Related Stigma and Discrimination against PLWHA in Nigerian Population. *PLoS One* 2015;10(12):e0143749. doi: 10.1371/journal.pone.0143749 [published Online First: 2015/12/15]
8. Toth S, J AY, DePinto N. HIV stigma: perceptions from HIV-positive and HIV-negative patients in a community dental clinic. *Journal of dental research, dental clinics, dental prospects* 2016;10(4):263-69. doi: 10.15171/joddd.2016.042 [published Online First: 2017/01/18]
9. Nyblade L, Stangl A, Weiss E, et al. Combating HIV stigma in health care settings: what works? *J Int AIDS Soc* 2009;12:15. doi: 10.1186/1758-2652-12-15 [published Online First: 2009/08/08]
10. Clark HJ, Lindner G, Armistead L, et al. Stigma, disclosure, and psychological functioning among HIV-infected and non-infected African-American women. *Women & health* 2004;38(4):57-71.
11. Lindberg MH, Wettergren L, Wiklander M, et al. Psychometric evaluation of the HIV stigma scale in a Swedish context. *PLoS One* 2014;9(12):e114867. doi: 10.1371/journal.pone.0114867 [published Online First: 2014/12/19]
12. Earnshaw VA, Chaudoir SR. From conceptualizing to measuring HIV stigma: a review of HIV stigma mechanism measures. *Aids Behav* 2009;13(6):1160-77. doi: 10.1007/s10461-009-9593-3 [published Online First: 2009/07/29]
13. Berger BE, Ferrans CE, Lashley FR. Measuring stigma in people with HIV: Psychometric assessment of the HIV stigma scale. *Research in nursing & health* 2001;24(6):518-29. doi: doi.org/10.1002/nur.10011
14. Reinius M, Wettergren L, Wiklander M, et al. Development of a 12-item short version of the HIV stigma scale. *Health and Quality of Life Outcomes* 2017;15(1):115. doi: 10.1186/s12955-017-0691-z [published Online First: 2017/06/01]
15. Kingori C, Reece M, Obeng S, et al. Psychometric evaluation of a cross-culturally adapted felt stigma questionnaire among people living with HIV in Kenya. *Aids*

For peer review only - http://bmjopen.bmj.com/site/about/guidelines.xhtml

Patient Care St 2013;27(8):481-8. doi: 10.1089/apc.2012.0403 [published Online First: 2013/08/24]
16. Wiklander M, Rydstrom LL, Ygge BM, et al. Psychometric properties of a short version of the HIV stigma scale, adapted for children with HIV infection. *Health Qual Life Outcomes* 2013;11(1):195. doi: 10.1186/1477-7525-11-195 [published Online First: 2013/11/15]
17. Holzemer WL, Uys LR, Chirwa ML, et al. Validation of the HIV/AIDS Stigma Instrument—PLWA (HASI-P). 2007;19(8):1002-12.
18. Bauman L, Silver E, Camacho S Stigma among mothers with HIV/AIDS. XIII International AIDS Conference, Durban, South Africa; 2000
19. Fife BL, Wright ER. The dimensionality of stigma: a comparison of its impact on the self of persons with HIV/AIDS and cancer. *J Health Soc Behav* 2000;41(1):50-67. [published Online First: 2000/04/06]
20. Herek GM, Capitanio JP. Public reactions to AIDS in the United States: a second decade of stigma. *Am J Public Health* 1993;83(4):574-7. doi: 10.2105/ajph.83.4.574 [published Online First: 1993/04/01]
21. Sowell RL, Seals BF, Moneyham L, et al. Quality of life in HIV-infected women in the south-eastern United States. *AIDS Care* 1997;9(5):501-12. doi: 10.1080/713613191 [published Online First: 1997/12/24]
22. Jeyaseelan L, Kumar S, Mohanraj R, et al. Assessing HIV/AIDS stigma in South India: validation and abridgement of the Berger HIV stigma scale. 2013;17(1):434-43.
23. Bunn JY, Solomon SE, Miller C, et al. Measurement of stigma in people with HIV: a reexamination of the HIV Stigma Scale. *AIDS Educ Prev* 2007;19(3):198-208. doi: 10.1521/aeap.2007.19.3.198 [published Online First: 2007/06/15]
24. Emlet CA, Brennan DJ, Brennenstuhl S, et al. The impact of HIV-related stigma on older and younger adults living with HIV disease: does age matter? *AIDS care* 2015;27(4):520-28.
25. Pannetier J, Lelievre E, Le Coeur S. HIV-related stigma experiences: Understanding gender disparities in Thailand. *AIDS Care* 2016;28(2):170-8. doi: 10.1080/09540121.2015.1096888 [published Online First: 2015/10/20]
26. Endeshaw M, Walson J, Rawlins S, et al. Stigma in Ethiopia: association with depressive symptoms in people with HIV. *AIDS Care* 2014;26(8):935-9. doi: 10.1080/09540121.2013.869537 [published Online First: 2014/01/03]
27. Datta S, Bhattacherjee S, Sherpa PL, et al. Perceived HIV Related Stigma among Patients Attending ART Center of a Tertiary Care Center in Rural West Bengal, India. *J Clin Diagn Res* 2016;10(10):VC09-VC12. doi: 10.7860/JCDR/2016/21366.8669 [published Online First: 2016/11/29]
28. Sorsdahl KR, Mall S, Stein DJ, et al. The prevalence and predictors of stigma amongst people living with HIV/AIDS in the Western Province. *AIDS Care* 2011;23(6):680-5. doi: 10.1080/09540121.2010.525621 [published Online First: 2011/03/02]
29. Yeo TED, Chu TH. Social-cultural factors of HIV-related stigma among the Chinese general population in Hong Kong. *AIDS Care* 2017;29(10):1255-59. doi: 10.1080/09540121.2017.1282601 [published Online First: 2017/01/24]
30. Cuca YP, Onono M, Bukusi E, et al. Factors associated with pregnant women's anticipations and experiences of HIV-related stigma in rural Kenya. *AIDS Care* 2012;24(9):1173-80. doi: 10.1080/09540121.2012.699669 [published Online First: 2012/07/18]
31. Girma E, Gebretsadik LA, Kaufman MR, et al. Stigma against people with HIV/AIDS in rural Ethiopia, 2005 to 2011: signs and predictors of improvement. *Aids Behav*

For peer review only - http://bmjopen.bmj.com/site/about/guidelines.xhtml

2014;18(6):1046-53. doi: 10.1007/s10461-013-0611-0 [published Online First: 2013/09/28]

32. Takada S, Weiser SD, Kumbakumba E, et al. The Dynamic Relationship Between Social Support and HIV-Related Stigma in Rural Uganda. *Ann Behav Med* 2014;48(1):26-37. doi: 10.1007/s12160-013-9576-5

33. Emlet CA. A comparison of HIV stigma and disclosure patterns between older and younger adults living with HIV/AIDS. *Aids Patient Care and Stds* 2006;20(5):350-58. doi: DOI 10.1089/apc.2006.20.350

34. Anakwa NO, Teye-Kwadjo E, Kretchy IA. Effect of HIV-Related Stigma and HIV-Related Stress on HIV Disclosure Concerns: a Study of HIV-Positive Persons on Antiretroviral Therapy at Two Urban Hospitals in Ghana. *Applied Research in Quality of Life* 2020:1-16.

35. Scott JA, Bauni E, Moisi JC, et al. Profile: The Kilifi Health and Demographic Surveillance System (KHDSS). *Int J Epidemiol* 2012;41(3):650-7. doi: 10.1093/ije/dys062 [published Online First: 2012/05/01]

36. Ngugi E, Kipruto S, Samoei P. Exploring Kenya's Inequality: Pulling Apart or Pooling Together? Kilifi County. Nairobi, Kenya: Kenya National Bureau of Statistics (KNBS) and the Society for International Development (SID) 2013.

37. Commission on Revenue Allocation (CRA). Kenya County Fact sheets: ; 2011 [Available from: http://siteresources.worldbank.org/INTAFRICA/Resources/257994-1335471959878/Kenya_County_Fact_Sheets_Dec2011.pdf accessed 22nd August 2018.

38. National AIDS Control Council. Kenya HIV County Profiles - 2016: National AIDS Control Council; 2016 [49-52]. Available from: http://nacc.or.ke/wp-content/uploads/2016/12/Kenya-HIV-County-Profiles-2016.pdf accessed 20th August 2018.

39. Harris PA, Taylor R, Minor BL, et al. The REDCap consortium: Building an international community of software platform partners. 2019;95:103208.

40. Harris PA, Taylor R, Thielke R, et al. Research electronic data capture (REDCap)—a metadata-driven methodology and workflow process for providing translational research informatics support. 2009;42(2):377-81.

41. Spitzer RL, Kroenke K, Williams JB. Validation and utility of a self-report version of PRIME-MD: the PHQ primary care study. Primary Care Evaluation of Mental Disorders. Patient Health Questionnaire. *JAMA* 1999;282(18):1737-44. doi: 10.1001/jama.282.18.1737 [published Online First: 1999/11/24]

42. Monahan PO, Shacham E, Reece M, et al. Validity/reliability of PHQ-9 and PHQ-2 depression scales among adults living with HIV/AIDS in western Kenya. *J Gen Intern Med* 2009;24(2):189-97. doi: 10.1007/s11606-008-0846-z [published Online First: 2008/11/26]

43. Spitzer RL, Kroenke K, Williams JB, et al. A brief measure for assessing generalized anxiety disorder: the GAD-7. *Arch Intern Med* 2006;166(10):1092-7. doi: 10.1001/archinte.166.10.1092 [published Online First: 2006/05/24]

44. Osborn TL, Venturo-Conerly KE, Wasil AR, et al. Depression and anxiety symptoms, social support, and demographic factors among Kenyan high school students. *Journal of Child Family Studies* 2019:1-12.

45. Nyongesa MK, Mwangala PN, Mwangi P, et al. Neurocognitive and mental health outcomes and association with quality of life among adults living with HIV: a cross-sectional focus on a low-literacy population from coastal Kenya. *Bmj Open* 2018;8(9):e023914. doi: 10.1136/bmjopen-2018-023914 [published Online First: 2018/09/19]

For peer review only - http://bmjopen.bmj.com/site/about/guidelines.xhtml

46. Filmer D, Pritchett LHJD. Estimating wealth effects without expenditure data—or tears: an application to educational enrollments in states of India. *%J Demography* 2001;38(1):115-32.
47. Nyongesa MK, Mwangi P, Wanjala SW, et al. Correlates of health-related quality of life among adults receiving combination antiretroviral therapy in coastal Kenya. *Health quality of life outcomes* 2020;18:1-14. doi: 10.1186/s12955-020-01421-0
48. Rosseel Y. Lavaan: An R package for structural equation modeling and more. Version 0.5–12 (BETA). *Journal of statistical software* 2012;48(2):1-36.
49. R Core Team. R: A language and environment for statistical computing. R Foundation for Statistical Computing. [computer program] 4.0.2 Vienna, Austria. 2020.
50. Blunch N. Introduction to structural equation modeling using IBM SPSS statistics and AMOS: Sage 2012.
51. Van de Schoot R, Lugtig P, Hox J. A checklist for testing measurement invariance. *European Journal of Developmental Psychology* 2012;9(4):486-92.
52. Cheung GW, Rensvold RB. Assessing extreme and acquiescence response sets in cross-cultural research using structural equations modeling. *Journal of cross-cultural psychology* 2000;31(2):187-212.
53. Ware Jr JE, Gandek BJJoce. Methods for testing data quality, scaling assumptions, and reliability: the IQOLA Project approach. 1998;51(11):945-52.
54. Cohen J. Statistical power analysis for the behavioural sciences, 2nd edn.(Hillsdale, NJ: L. Erlbaum Associates). 1988
55. Tabachnik B, Fidell L. Using multivariate statistics (6e éd.). Boston, É.-U. *U: Pearson* 2013
56. Terwee CB, Bot SD, de Boer MR, et al. Quality criteria were proposed for measurement properties of health status questionnaires. *Journal of clinical epidemiology* 2007;60(1):34-42. doi: 10.1016/j.jclinepi.2006.03.012
57. Streiner DL. A checklist for evaluating the usefulness of rating scales. *Canadian journal of psychiatry Revue canadienne de psychiatrie* 1993;38(2):140-8. doi: 10.1177/070674379303800214 [published Online First: 1993/03/01]
58. StataCorp LP. Stata statistical software: release 14. [computer program] College Station, TX; StataCorp LP: 2015.
59. Akena D, Joska J, Obuku EA, et al. Sensitivity and specificity of clinician administered screening instruments in detecting depression among HIV-positive individuals in Uganda. *AIDS Care* 2013;25(10):1245-52. doi: 10.1080/09540121.2013.764385 [published Online First: 2013/02/13]
60. Gadermann A, Guhn M, Zumbo B. Estimating ordinal reliability for Likert-type and ordinal item response data: A conceptual, empirical, and practical guide. P Ass, Re & Evalu, 17 (3), 1-13, 2012.
61. González J, Pazmiño MJRP. Cálculo e interpretación del Alfa de Cálculo e interpretación del Alfa de Cronbach para el caso de validación de la consistencia interna de un cuestionario, con dos posibles escalas tipo Likert. 2015;2(1):62-77.
62. Jeyaseelan L, Kumar S, Mohanraj R, et al. Assessing HIV/AIDS stigma in south India: validation and abridgement of the Berger HIV Stigma scale. *Aids Behav* 2013;17(1):434-43. doi: 10.1007/s10461-011-0128-3 [published Online First: 2012/01/17]
63. Nattabi B, Li J, Thompson SC, et al. Factors associated with perceived stigma among people living with HIV/AIDS in post-conflict northern Uganda. *AIDS Education and Prevention* 2011;23(3):193-205.

For peer review only - http://bmjopen.bmj.com/site/about/guidelines.xhtml

64. Swendeman D, Rotheram-Borus MJ, Comulada S, et al. Predictors of HIV-related stigma among young people living with HIV. *Health Psychol* 2006;25(4):501-9. doi: 10.1037/0278-6133.25.4.501 [published Online First: 2006/07/19]
65. Asiedu GB, Myers-Bowman KS. Gender differences in the experiences of HIV/AIDS-related stigma: a qualitative study in Ghana. *Health Care Women Int* 2014;35(7-9):703-27. doi: 10.1080/07399332.2014.895367 [published Online First: 2014/02/26]
66. Mugoya GC, Ernst K. Gender differences in HIV-related stigma in Kenya. *AIDS Care* 2014;26(2):206-13. doi: 10.1080/09540121.2013.808733 [published Online First: 2013/06/26]
67. Mbonu NC, van den Borne B, De Vries NK. Stigma of People with HIV/AIDS in Sub-Saharan Africa: A Literature Review. *J Trop Med* 2009;2009:145891. doi: 10.1155/2009/145891 [published Online First: 2009/01/01]
68. Amuyunzu-Nyamongo M, Okeng'o L, Wagura A, et al. Putting on a brave face: the experiences of women living with HIV and AIDS in informal settlements of Nairobi, Kenya. *AIDS Care* 2007;19 Suppl 1(sup1):S25-34. doi: 10.1080/09540120601114618 [published Online First: 2007/03/17]
69. Muyinda H, Seeley J, Pickering H, et al. Social aspects of AIDS-related stigma in rural Uganda. 1997;3(3):143-47.
70. Linda P. To tell or not to tell: negotiating disclosure for people living with HIV on antiretroviral treatment in a South African setting. *SAHARA J* 2013;10 Suppl 1(sup1):S17-27. doi: 10.1080/02664763.2012.755320 [published Online First: 2013/07/17]
71. Evangeli M, Wroe AL. HIV Disclosure Anxiety: A Systematic Review and Theoretical Synthesis. *Aids Behav* 2017;21(1):1-11. doi: 10.1007/s10461-016-1453-3 [published Online First: 2016/07/14]
72. Nyongesa MK, Mwangi P, Wanjala SW, et al. Prevalence and correlates of depressive symptoms among adults living with HIV in rural Kilifi, Kenya. *BMC Psychiatry* 2019;19(1):333. doi: 10.1186/s12888-019-2339-5 [published Online First: 2019/11/05]
73. Charles B, Jeyaseelan L, Pandian AK, et al. Association between stigma, depression and quality of life of people living with HIV/AIDS (PLHA) in South India–a community based cross sectional study. 2012;12(1):463.
74. Yi S, Chhoun P, Suong S, et al. AIDS-related stigma and mental disorders among people living with HIV: a cross-sectional study in Cambodia. 2015;10(3):e0121461.
75. Liu Y, Gong H, Yang G, et al. Perceived stigma, mental health and unsafe sexual behaviors of people living with HIV/AIDS. 2014;39(7):658-63.
76. Onyebuchi-Iwudibia O, Brown A. HIV and depression in Eastern Nigeria: the role of HIV-related stigma. *AIDS Care* 2014;26(5):653-7. doi: 10.1080/09540121.2013.844761 [published Online First: 2013/10/19]
77. Oke OO, Akinboro AO, Olanrewaju FO, et al. Assessment of HIV-related stigma and determinants among people living with HIV/AIDS in Abeokuta, Nigeria: A cross-sectional study. *SAGE Open Medicine* 2019;7:2050312119869109.
78. Galvan FH, Davis EM, Banks D, et al. HIV stigma and social support among African Americans. 2008;22(5):423-36.
79. Stephenson R. Community factors shaping HIV-related stigma among young people in three African countries. *AIDS care* 2009;21(4):403-10.
80. Cianelli R, Villlegas N, De Oliveira G, et al. Predictors of HIV enacted stigma among Chilean women. 2015;24(17-18):2392-401.
81. Li Z, Morano JP, Khoshnood K, et al. HIV-related stigma among people living with HIV/AIDS in rural Central China. 2018;18(1):453.

For peer review only - http://bmjopen.bmj.com/site/about/guidelines.xhtml

82. Chan BT, Tsai AC, Siedner MJ. HIV Treatment Scale-Up and HIV-Related Stigma in Sub-Saharan Africa: A Longitudinal Cross-Country Analysis. *Am J Public Health* 2015;105(8):1581-7. doi: 10.2105/AJPH.2015.302716 [published Online First: 2015/06/13]

83. Wolfe WR, Weiser SD, Leiter K, et al. The impact of universal access to antiretroviral therapy on HIV stigma in Botswana. *Am J Public Health* 2008;98(10):1865-71. doi: 10.2105/AJPH.2007.122044 [published Online First: 2008/08/16]

84. Jurgensen M, Sandoy IF, Michelo C, et al. Effects of home-based voluntary counselling and testing on HIV-related stigma: findings from a cluster-randomized trial in Zambia. *Soc Sci Med* 2013;81:18-25. doi: 10.1016/j.socscimed.2013.01.011 [published Online First: 2013/02/21]

85. Kemp CG, Lipira LL, David H, et al. HIV stigma and viral load among African-American women receiving treatment for HIV: A longitudinal analysis. 2019;33(9):1511.

For peer review only

Table 1: Participant's sociodemographic characteristics

**Sample Characteristics**	**Total sample N=450**	**%**
**Sociodemographic characteristics**		
Age – years Range (18-60), Median (IQR)	43(14)	
Gender		
Female	356	79.1
Male	94	20.9
Marital Status		
Married/cohabiting	196	43.6
Separated /Divorced/Widowed	197	43.8
Single/Never Married	57	12.7
Education		
Tertiary	22	4.9
Secondary	66	14.7
Primary	239	53.1
None	123	27.3
Employment		
Formally employed	53	11.8
Self-employed	117	26.0
Other	11	2.4
Unemployed (including students)	269	59.8
Currently living with		
Family	371	82.4
Relative/friend	10	2.2
Alone	69	15.3
Asset index score [a] – mean (SD)	1.2(1.4)	1.4
Perceived HIV-stigma score [b] – mean (SD)	28.4(7.7)	7.7
Any current chronic illness		
No	413	91.8
Yes	37	8.2
**Clinical characteristics**		
BMI – kg/m2, mean (SD), OM = 4	22.4 (4.8)	
cART regimen, OM = 4		
First-line	425	95.3
Second line	21	4.7
Viral load, OM = 145		
≤ 1000 copies/mL	265	86.9
> 1000 copies/mL	40	13.1
WHO clinical stage, OM = 5		
Stage 1	417	93.7
Stage 2	22	4.9
Stage 3	3	0.7
Stage 4	3	0.7
Months since HIV diagnosis – Median (IQR)	106 (82)	
Months since cART initiation – Median (IQR)	80.5 (76)	
**Treatment Characteristics**		
HIV status disclosure		
Yes	423	94.0
No	27	6.0
Any current opportunistic infection		
No	367	81.6
Yes	83	18.4

Notes: OM = Observation with missing value, SD = Standard deviation, a - score range = 0 to 7, b – score range = 12 to 48, IQR = Interquartile range

For peer review only - http://bmjopen.bmj.com/site/about/guidelines.xhtml

For peer review only

Table 2: Descriptive statistics for items and subscales in the short form 12-item Swahili version of the HIV Stigma Scale

Item	Mean item score[a] (SD)	Corrected item correlation	Mean subscale score[b] (SD)	Reliability: Internal consistency (Cronbach α)	Reliability: Test-Retest (ICC)	Validity: Convergent ≠	Validity: Convergent #	Validity: Construct CFI	Validity: Construct RMSEA	Validity: Construct TLI
**Personalised Stigma**			4.86(2.56)	0.84 (95% CI; 0.81-0.86)	0.83 (95% CI; 0.71-0.90)	0.357**	0.327**			
Some people stop touching me soon they know/realise I am infected with HIV/AIDS	1.66(1.01)	0.65								
People I care for stopped calling me after knowing I suffer from AIDs.	1.63(1.00)	0.87								
I have lost friends for telling/explaining that I have AIDs.	1.59(0.96)	0.88								
**Disclosure Concerns**			8.74(2.37)	0.53 (95% CI; 0.45-0.60)	0.62 (95% CI; 0.36-0.77)	0.070	0.070			
Telling someone that I have AIDs is dangerous*	2.24(1.24)	0.83								
I do all I can to keep my AIDs (HIV) status secret	2.90(1.22)	0.46								
I am very careful to that person I tell about my HIV status (I am cautious/ very careful to (?of) the people I tell my HIV status)	3.60(0.78)	0.21								
**Concerns about Public Attitudes**			8.52(3.17)	0.83 (95% CI; 0.80-0.86)	0.79 (95% CI; 0.65-0.88)	0.187**	0.165**			
People who are suffering from AIDs are treated as if they are not like the other people.	3.05(1.18)	0.68								
People believe that a person infected with HIV is dirty.	2.74(1.26)	0.84								
Many people are worried when they are near a person infected with HIV.	2.75(1.22)	0.84								
**Negative Self Image**			6.32(3.00)	0.74 (95% CI; 0.70-0.80)	0.76 (95% CI; 0.60-0.86)	0.372**	0.330**			
I feel guilty because I am infected with HIV	2.11(1.23)	0.60								
People's attitudes about HIV/AIDs makes me feel very bad.	2.23(1.25)	0.78								
I feel I am not as good as others because I'm infected with HIV.	2.01(1.23)	0.73								
**Overall**			28.44(7.68)	0.80(95% CI; 0.77-0.83)	0.92(95% CI; 0.87-0.95)	0.368**	0.328**	0.966	0.044	0.955

Pearson product-moment correlation coefficient; **p<0.001; # correlation between HIV stigma and PHQ-9; ≠ correlation between HIV stigma and GAD-7
[a]Possible score for each item 1-4; higher scores reflect a higher level of perceived HIV-related stigma
[b]Possible score 3-12 on each sub-scale; higher scores reflect a higher level of perceived HIV- related stigma. [SD] Standard deviation. CFI = Comparative Fit Index. RMSEA = Root Mean Square Error of Approximation and TLI=Tucker Lewis Index. Cronbach α- Cronbach alpha

For peer review only - http://bmjopen.bmj.com/site/about/guidelines.xhtml

Table 3: Univariate linear Regression of correlates of perceived HIV-related stigma among adults living with HIV from rural Kilifi

Independent variables	N	Dependent variables: Personalised Stigma		Disclosure concerns		Public attitudes		Negative self-image		Overall HIV Stigma Score	
		B (95% CI)	p-value	B (95% CI)	p-value	B (95% CI)	p-value	B (95% CI)	p-value	B (95% CI)	p-value
**Sociodemographic characteristics**											
Age	450	-0.01 (-0.03, 0.02)	0.595	0.01 (-0.02, 0.02)	0.999	0.01 (-0.02, 0.04)	0.399	-0.01(-0.03, 0.03)	0.880	0.01 (-0.07, 0.08)	0.910
Gender	450										
Male		Ref		Ref		Ref		Ref		Ref	
Female		0.52(-0.06, 1.10)	0.080*	0.29 (-0.25, 0.83)		1.07 (0.35, 1.79)	0.003**	0.40 (-0.29, 1.08)	0.255	2.27 (0.54, 4.01)	0.010**
Marital Status	450		0.074								
Married		Ref		Ref		Ref		Ref		Ref	
Separated/Divorced/Widowed		0.54(0.03, 1.04)	0.038**	0.67(0.20, 1.14)	0.005**	0.25(-0.38, 0.87)	0.442	0.73(0.14, 1.32)	0.016**	2.18(0.67, 3.69)	0.005**
Single/never married		0.61(-0.14, 1.37)	0.111*	0.17 (-0.52, 0.87)	0.626	-0.43(-1.37, -0.51)	0.369	0.40 (-0.49, 1.28)	0.378	0.75(-1.50, 3.01)	0.512
Education Level	450										
Tertiary		Ref		Ref		Ref		Ref		Ref	
Secondary		-0.12(-1.36, 1.12)	0.847	0.08 (-1.06, 1.21)	0.896	0.68(-0.84, 2.21)	0.380	-0.03 (-1.46, 1.40)	0.967	0.61(-3.05, 4.26)	0.745
Primary		-0.31(-1.43, 0.81)	0.582	0.48 (-0.55, 1.51)	0.360	1.32(-0.06, 2.70)	0.061*	0.72 (-0.57, 2.01)	0.273	2.20(-1.10, 5.51)	0.191*
None		0.15(-1.01, 1.32)	0.794	1.23 (0.16, 2.30)	0.024**	1.80(0.36, 3.23)	0.014**	1.63 (0.29, 2.97)	0.018**	4.81(1.38, 8.25)	0.006**
Employment Status	450										
Formally Employed		Ref		Ref		Ref		Ref		Ref	
Self-employed		0.67(-0.16, 1.50)	0.112*	0.27 (-0.50, 1.05)	0.490	0.46(-0.57, 1.49)	0.385	0.73*(-0.24, 1.70)	0.141*	2.13(-0.36, 4.62)	0.094*
Other		-0.67(-2.33, 0.99)	0.429	-0.02 (-1.57, 1.53)	0.983	-1.14(-3.20, 0.93)	0.279	-0.35 (-2.29, 1.60)	0.726	-2.17(-7.15, 2.81)	0.392
Unemployed		0.51(-0.25, 1.26)	0.187*	0.33 (-0.37, 1.03)	0.360	0.18(-0.76, 1.11)	0.710	1.03 (0.15, 1.91)	0.022**	2.04(-0.22, 4.30)	0.077*
Currently living with	450										
Immediate family		Ref		Ref		Ref		Ref		Ref	
Relative/friend		0.86(-0.75, 2.47)	0.294	0.02(-1.47, 1.52)	0.975	0.18(-1.82, 2.18)	0.862	-0.45(-2.34,1.45)	0.644	0.62(-4.23, 5.46)	0.802
Alone		0.01(-0.65, 0.66)	0.995	-0.25(-0.87, 0.36)	0.414	-0.06(-0.88, 0.76)	0.887	-0.07(-0.84, 0.70)	0.860	-0.38(-2.36, 1.60)	0.706
Asset index score [a] – mean (SD)	450	-0.12(-0.29-0.05)	0.171*	-0.13(-0.29-0.03)	0.109*	-0.11(-0.33-0.10)	0.310	-0.12 (-0.32-0.08)	0.244*	-0.48(-1.00-0.04)	0.068*
Clinical characteristics											
BMI – kg/m^2, mean (SD), OM = 4		0.004(-0.04, 0.05)	0.855	-0.03(-0.07,0.02)	0.244	0.03(-0.03,0.09)	0.309	-0.03(-0.18,0.12)	0.708	-0.03(-0.18,0.12)	0.708
Viral Load OM = 145	305										
≤ 1000 copies/ml		Ref		Ref		Ref		Ref		Ref	
> 1000 copies/ml		0.58(-0.28, 1.44)	0.183*	0.10(-0.70, 0.90)		0.07(-1.00, 1.14)	0.894	1.05 (0.08,2.02)	0.033**	1.81(-0.79, 4.40)	0.172*

For peer review only - http://bmjopen.bmj.com/site/about/guidelines.xhtml

Months since HIV diagnosis	450	0.00(-0.00, 0.01)	0.346	-0.01 (-0.01, -0.00)	0.001**	-0.00(-0.01,0.00)	0.630	-0.01 (-0.01,0.00)	0.057*	-0.01(0.03,0.00)	0.091*
Months since cART initiation OM = 4	446	0.00(-0.00,0.01)	0.497	-0.01 (-0.01, -0.00)	0.001***	-0.00(-0.01,0.00)	0.202*	-0.01 (-0.01, -0.00)	0.031**	-0.02(-0.03, -0.00)	0.031**
**Treatment characteristics**											
HIV status disclosure	450										
Yes		Ref		Ref		Ref		Ref		Ref	
No		0.23(-0.77,1.23)	0.651	1.86 (0.94,2.77)	0.000***	0.67(-0.57,1.91)	0.287	0.72(-0.45,1.89)	0.228	3.47(0.49,6.46)	0.022**
Any current opportunistic infections	450										
No		Ref		Ref		Ref		Ref		Ref	
Yes		0.65(0.04,1.26)	0.037**	0.09(-0.48,0.65)	0.786	0.12(-0.64,0.88)	0.759	0.87 (0.16,1.59)	0.017**	1.72(-0.11,3.55)	0.065*
CMD comorbidity OM = 48	402										
Absence		Ref		Ref		Ref		Ref		Ref	
Presence		2.71(1.58, 3.84)	0.000***	0.18 (-0.91, 1.28)	0.741	1.09(-0.38, 2.55)	0.144*	3.07 (1.76, 4.39)	0.000***	7.06(3.71,10.41)	0.000***

Notes: Overall stigma scale represents the sum of all twelve items from the four subscales; A negative stigma score indicates less stigma**.** *CMD* – symptoms of depression and anxiety, *BMI* body mass index, *WHO* World Health Organisation, *Ref* - Reference category, *OM* observation with missing value, cART combination antiretroviral therapy, *a* score range = 0 to 7, *b* score range = 12 to 48. * p ≤0.25; **p<0.05; ***p<0.001

For peer review only

Table 4: Multivariate linear Regression of correlates of perceived HIV-related stigma among adults living with HIV from rural Kilifi

**Independent variables**	**Dependent variables**									
	**Personalised stigma (n=402)**		**Disclosure concerns (n=450)**		**Public attitudes (n=450)**		**Negative self-image (n=402)**		**Overall HIV Stigma Score (n=402)**	
	**B (95% CI)**	**p-value**	**B (95% CI)**	**p-value**	**B (95% CI)**	**p-value**	**B (95% CI)**	**p-value**	**B (95% CI)**	**p-value**
**Sociodemographic characteristics**										
Gender										
Male	Ref				Ref				Ref	
Female	0.75(0.17, 1.34)	0.012**			1.07(0.35,1.79)	0.003**			2.00(0.21,3.80)	0.029**
Education Level										
Tertiary			Ref				Ref		Ref	
Secondary			-0.04(-1.14,1.07)	0.950			-0.05(-1.44,1.33)	0.939	-0.34(-3.83,3.16)	0.850
Primary			0.48(-0.52,1.48)	0.346			0.51(-0.73,1.74)	0.423	1.37(-1.75,4.50)	0.388
None			1.24(0.20,2.28)	0.019**			1.33(0.04, 2.62)	0.044**	3.32(-0.01,6.65)	0.051
**Clinical characteristics**										
Months since HIV diagnosis			-0.01(-0.01, -0.00)	0.007**						
**Treatment characteristics**										
HIV status disclosure										
Yes			Ref						Ref	
No			1.79(0.88,2.70)	0.000***					4.24(1.27,7.20)	0.005**
CMD comorbidity										
Absence	Ref						Ref		Ref	
Presence	2.67(1.55, 3.79)	0.000***					3.04(1.74, 4.34)	0.000***	6.67(3.40,9.94)	0.000***
Variance explained by the model Pseudo R-squared	6.76%		8.66%		1.89%		7.71%		10.17%	

Notes: Overall stigma scale represents the sum of all twelve items from the four sub-scales. **CMD** – symptoms of depression and anxiety, 95% CI - 95% confidence interval, Ref-reference category **p<0.05, ***p<0.001

For peer review only

Table 5: Multi-Group Confirmatory Factor Analysis for age and gender sub-groups

Invariance Steps	Gender	RMSEA	TLI	CFI	ΔCFI	Age	RMSEA	TLI	CFI	ΔCFI
Configural Invariance	Female	0.051	0.934	0.950		Older adults	0.040	0.960	0.970	
	Male	0.051	0.934	0.950		Young Adults	0.040	0.960	0.970	
Metric Invariance	Female	0.052	0.932	0.943	0.007	Older adults	0.042	0.957	0.964	0.006
	Male	0.052	0.932	0.943	0.007	Young Adults	0.042	0.957	0.964	0.006
Scalar Invariance	Female	0.050	0.936	0.943	0.000	Older adults	0.041	0.959	0.963	0.001
	Male	0.050	0.936	0.943	0.000	Young Adults	0.041	0.959	0.963	0.001
Strict Invariance	Female	0.048	0.941	0.942	0.001	Older adults	0.041	0.959	0.960	0.003
	Male	0.048	0.941	0.942	0.001	Young Adults	0.041	0.959	0.960	0.003

Notes: Criteria for an acceptable fit were a root mean square error of approximation (RMSEA) of < 0.06, and a comparative fit index (CFI), and a Tucker-Lewis index (TLI) of ≥0.90. Configural invariance - no constraints; Full metric invariance - with all factor loadings constrained equal. Scalar invariance - with all intercepts constrained equal; Strict invariance – with all factor loadings and intercepts fixed; Measurement invariance is assumed when ΔCFI is ≤0.01

Figure Legends

Supplementary Figure 1: Confirmatory factor analysis of the short version of the HIV Stigma Scale. Results show correlations between subscales (circles) and maximum likelihood estimates for the relation between subscales and items (rectangles). Sample (n = 435). Maximum likelihood estimates are standardised

Figure 1: Study recruitment flow chart

For peer review only - http://bmjopen.bmj.com/site/about/guidelines.xhtml

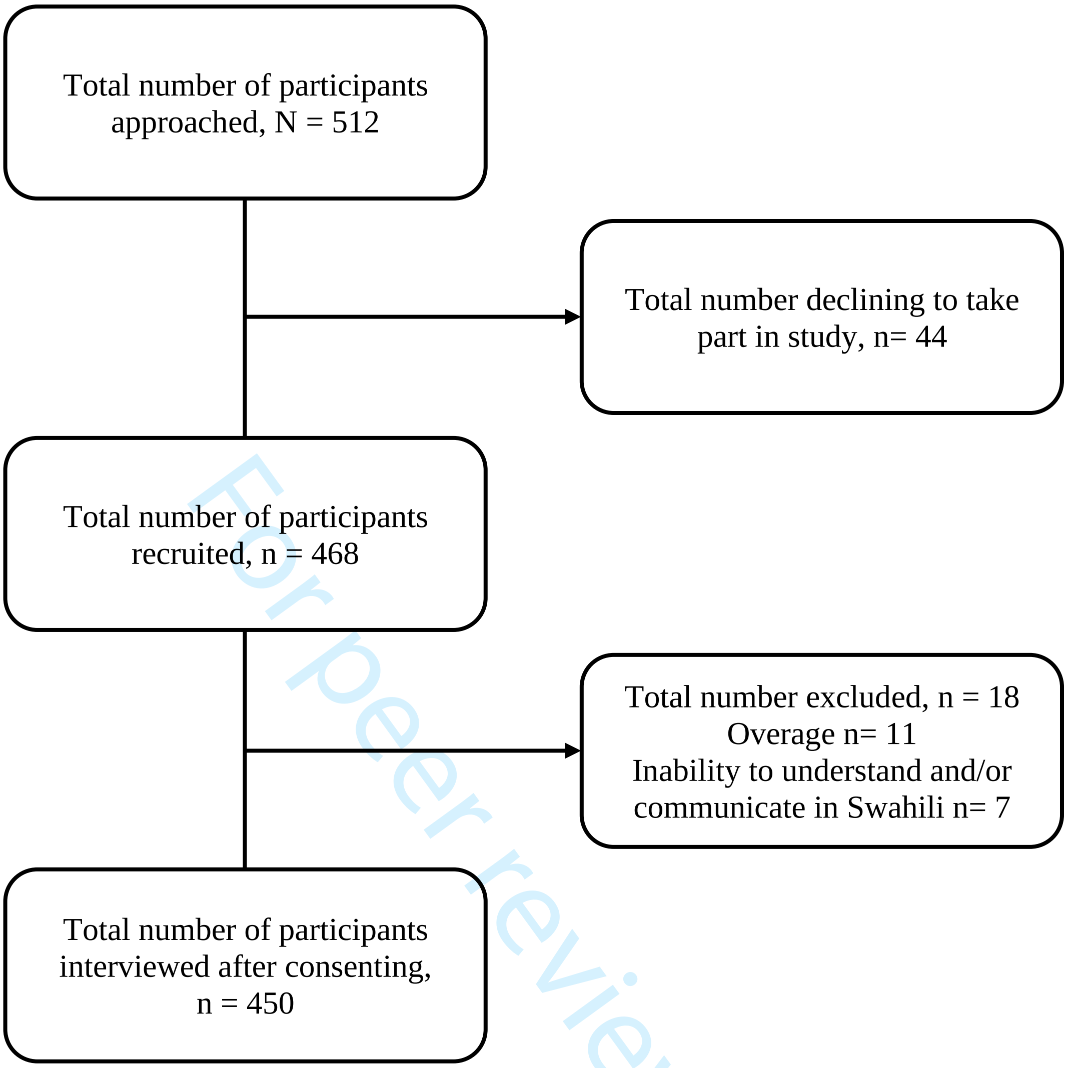

Figure 1: Study recruitment flow chart

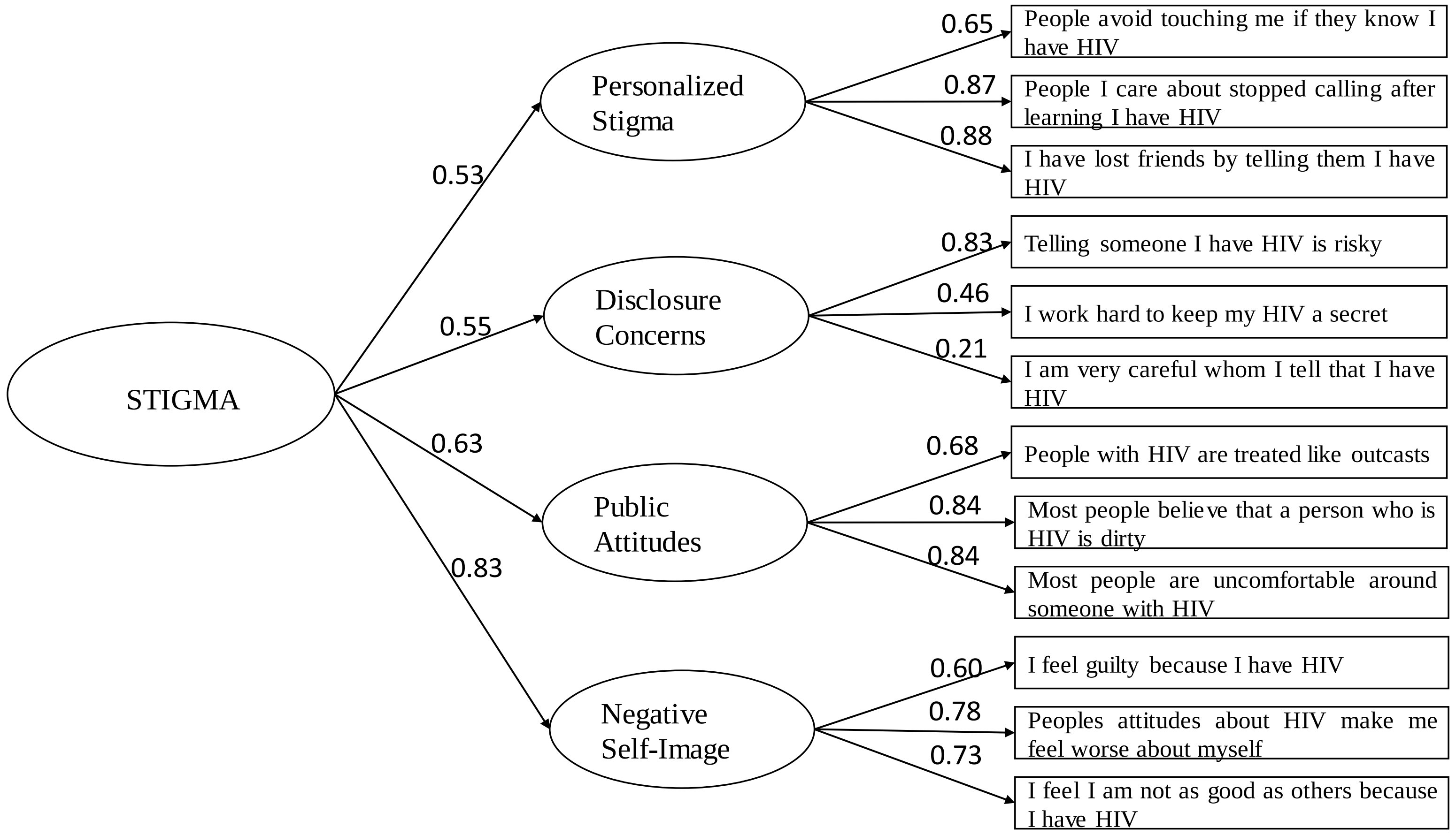

Supplementary Figure 1: Confirmatory factor analysis of the short version of the HIV Stigma Scale. Results show correlations between subscales (circles) and maximum likelihood estimates for the relation between subscales and items (rectangles). Sample (n = 435). Maximum likelihood estimates are standardised

STROBE 2007 (v4) Statement—Checklist of items that should be included in reports of *cross-sectional studies*

Section/Topic	Item #	Recommendation	Reported on page #
**Title and abstract**	1	(*a*) Indicate the study's design with a commonly used term in the title or the abstract	2
		(*b*) Provide in the abstract an informative and balanced summary of what was done and what was found	2
**Introduction**			
Background/rationale	2	Explain the scientific background and rationale for the investigation being reported	4-5
Objectives	3	State specific objectives, including any prespecified hypotheses	5
**Methods**			
Study design	4	Present key elements of study design early in the paper	5
Setting	5	Describe the setting, locations, and relevant dates, including periods of recruitment, exposure, follow-up, and data collection	5-8
Participants	6	(*a*) Give the eligibility criteria, and the sources and methods of selection of participants	6
Variables	7	Clearly define all outcomes, exposures, predictors, potential confounders, and effect modifiers. Give diagnostic criteria, if applicable	6-8
Data sources/ measurement	8*	For each variable of interest, give sources of data and details of methods of assessment (measurement). Describe comparability of assessment methods if there is more than one group	6-8
Bias	9	Describe any efforts to address potential sources of bias	6&8
Study size	10	Explain how the study size was arrived at	6
Quantitative variables	11	Explain how quantitative variables were handled in the analyses. If applicable, describe which groupings were chosen and why	9
Statistical methods	12	(*a*) Describe all statistical methods, including those used to control for confounding	8-10
		(*b*) Describe any methods used to examine subgroups and interactions	N/A
		(*c*) Explain how missing data were addressed	N/A
		(*d*) If applicable, describe analytical methods taking account of sampling strategy	8-9
		(*e*) Describe any sensitivity analyses	N/A
**Results**			

For peer review only

Participants	13*	(a) Report numbers of individuals at each stage of study—eg numbers potentially eligible, examined for eligibility, confirmed eligible, included in the study, completing follow-up, and analysed	29
		(b) Give reasons for non-participation at each stage - see details in figure 1	29
		(c) Consider use of a flow diagram	29
Descriptive data	14*	(a) Give characteristics of study participants (eg demographic, clinical, social) and information on exposures and potential confounders	10
		(b) Indicate number of participants with missing data for each variable of interest	23
Outcome data	15*	Report numbers of outcome events or summary measures	
Main results	16	(*a*) Give unadjusted estimates and, if applicable, confounder-adjusted estimates and their precision (eg, 95% confidence interval). Make clear which confounders were adjusted for and why they were included	25-27
		(*b*) Report category boundaries when continuous variables were categorized - see details in Table 1	23
		(*c*) If relevant, consider translating estimates of relative risk into absolute risk for a meaningful time period	N/A
Other analyses	17	Report other analyses done—eg analyses of subgroups and interactions, and sensitivity analyses	N/A
**Discussion**			
Key results	18	Summarise key results with reference to study objectives	10
Limitations	19	Discuss limitations of the study, taking into account sources of potential bias or imprecision. Discuss both direction and magnitude of any potential bias	15
Interpretation	20	Give a cautious overall interpretation of results considering objectives, limitations, multiplicity of analyses, results from similar studies, and other relevant evidence	12-15
Generalisability	21	Discuss the generalisability (external validity) of the study results	15
**Other information**			
Funding	22	Give the source of funding and the role of the funders for the present study and, if applicable, for the original study on which the present article is based	16

*Give information separately for cases and controls in case-control studies and, if applicable, for exposed and unexposed groups in cohort and cross-sectional studies.

Note: An Explanation and Elaboration article discusses each checklist item and gives methodological background and published examples of transparent reporting. The STROBE checklist is best used in conjunction with this article (freely available on the Web sites of PLoS Medicine at http://www.plosmedicine.org/, Annals of Internal Medicine at http://www.annals.org/, and Epidemiology at http://www.epidem.com/). Information on the STROBE Initiative is available at www.strobe-statement.org.

For peer review only
